# Integrative transcriptome, proteome, and microRNA analysis reveals the effects of nitrogen sufficiency and deficiency conditions on theanine metabolism in the tea plant (*Camellia sinensis*)

**DOI:** 10.1038/s41438-020-0290-8

**Published:** 2020-05-01

**Authors:** Zhi-Wei Liu, Hui Li, Jie-Xia Liu, Yu Wang, Jing Zhuang

**Affiliations:** 1 Tea Science Research Institute, Ministry of Agriculture and Rural Affairs Key Laboratory of Biology and Germplasm Enhancement of Horticultural Crops in East China, College of Horticulture, Nanjing Agricultural University, 210095 Nanjing, China; 2 State Key Laboratory of Crop Genetics and Germplasm Enhancement, Nanjing Agricultural University, 210095 Nanjing, China

**Keywords:** Secondary metabolism, Metabolism

## Abstract

Nitrogen (N) is associated with amino acid metabolism in higher plants. Theanine is an important amino acid in tea plants. To explore the relationship between theanine metabolism and N conditions, we examined the differentially expressed genes (DEGs), proteins (DEPs), and microRNAs (DEMs) involved in theanine metabolism in tea plant shoots and roots under N sufficiency and deficiency conditions. Transcriptome, proteome, and microRNA analyses were performed on tea plant shoots and roots under N sufficiency and deficiency conditions. The contents of theanine, expression levels of genes involved in theanine metabolism, contents of proteinogenic amino acids, and activity of enzymes were analyzed. The DEP–DEG correlation pairs and negative DEM–DEG interactions related to theanine metabolism were identified based on correlation analyses. The expression profiles of DEGs and negative DEM–DEG pairs related to theanine biosynthesis were consistent with the sequencing results. Our results suggest that the molecular and physiological mechanism of theanine accumulation is significantly affected by N sufficiency and deficiency conditions. The DEGs, DEPs, and DEMs and the activity of the enzymes involved in theanine biosynthesis might play vital roles in theanine accumulation under N sufficiency and deficiency conditions in the shoots and roots of tea plants.

## Introduction

In higher plants, amino acid metabolism, a type of nitrogen (N) metabolism, is an important primary metabolic process like that for carbon, fat, and nucleic acids^1^. The tea plant (*Camellia sinensis* (L.) O. Kuntze) is an important beverage crop that contains abundant free amino acids in fresh leaves^2^. Theanine, closely related to tea flavor and quality, is a unique free amino acid in tea plants^3,4^. The tissue specificity of theanine synthesis and distribution has been demonstrated by previous studies. Theanine can be synthesized in every part but is mainly synthesized in the roots of tea plants and shows the highest levels in shoots^5,6^. Due to the unique theanine metabolism pathway, N metabolism in tea plants is different from that in other plants^7,8^. The different forms and levels of inorganic N in soils influence theanine biosynthesis and accumulation. The roots of tea plants show a preference for ammonium (NH_4_^+^) compared with nitrate (NO_3_^−^), and NH_4_^+^ is more readily assimilated than NO_3_^−^ into theanine^7,9,10^. The main storage form of ammonium is arginine in tea plants when the carbon/nitrogen (C/N) ratio is low. It is theanine when the C/N value is suitable for tea plants^11^. The theanine contents in buds will increase several fold when tea plants grown under conditions of N deficiency are fertilized with N^12^.

Thus far, theanine synthase (TS), glutamine synthase (GS), glutamate synthase (GOGAT), glutamate dehydrogenase (GDH), alanine transaminase (ALT), l-alanine decarboxylase (AIDA), theanine hydrolase (ThYD) and amine oxidase (AO) have been confirmed to directly participate in theanine synthesis and hydrolysis^2,13,14^. In addition, several proteins or enzymes related to intermediates of the theanine or N metabolism pathway also play important roles in theanine accumulation. For example, nitrate reductase (NR), nitrite reductase (NiR), nitrate transporter (NRT), nitrogen regulatory protein P-II (GlnB), ammonium transporter (AMT) and aquaporin protein (AQP) are involved in N uptake and assimilation^15^. The glutamate receptor (GLR) has potential functions in regulating C/N balance^16^, and glutamate decarboxylase (GAD) is used for glutamate consumption^17^. GMP synthase (GMPS) and asparagine synthase (AS) hydrolyze glutamine, argininosuccinate synthase (ASS) synthesizes arginine, and phosphoenolpyruvate carboxylase (PEPC) accelerate the production of carbon skeletons for amino acid synthesis^18–20^. Moreover, pyruvate kinase (PK) is indispensable for pyruvate generation^21^, and pyruvate decarboxylase (PDC), pyruvate dehydrogenase (PDH) and the PDH-regulating enzymes pyruvate dehydrogenase kinase (PDK) and pyruvate dehydrogenase phosphatase (PDP) are involved in pyruvate consumption^22^. Enzymological research on most of these enzymes in tea plants still has been limited to analysis and determination of crude enzyme solutions; there have been few studies on the enzyme genes at the level of molecular biology.

With the development of sequencing technology, increasing amounts of tea plant transcriptome, proteome, and microRNA (miRNA) data have been made public^23^. Transcriptome analysis has been applied in tea plants to discover and analyze genes related to secondary metabolism, growth and development, and abiotic and biotic stress responses^24–35^. Via RNA-seq, the genes involved in theanine metabolism have been partially identified^14,24,36^. The expression profiles of genes involved in theanine metabolism have also been analyzed in different cultivars and tissues under drought and different light treatments^13,27,37^. Based on annotation of the recently published tea tree genome, homologous genes of *TS*, *GS*, *GDH*, *ADC*, *SAMDC*, and *Fe-GOGAT* have been obtained from the transcriptomes of 23 *Camellia* species^38^. Furthermore, the differential expression profiles of genes in the theanine biosynthesis pathway have been analyzed by integrated transcriptomic and biochemical, proteomic, and metabolic analyses^2,15,29,39–41^. The importance of CsGOGAT in theanine synthesis has been demonstrated in postharvest tea plant leaves through proteome analysis^42^. In addition, miRNAs involved in the regulation of bud dormancy, abiotic stress, and catechin accumulation have been discovered but are not associated with theanine metabolism^43–46^.

Tea plants in practical planting areas often suffer from N deficiency in soil. N deficiency leads to negative influences on tea quality due to reduced amino acid content, which largely determines the umami taste of tea^10^. In this study, we performed N deficiency treatment on tea plant seedlings cultivated in nutrient solution. Based on an omics research strategy, the differentially expressed genes (DEGs), proteins (DEPs), and miRNAs (DEMs) involved in theanine biosynthesis between tea plant shoots and roots under N sufficiency and deficiency treatments were screened from transcriptome, proteome, and miRNA data, respectively. In addition, the activity of five key enzymes related to theanine synthesis and the levels of 16 free proteinogenic amino acids were determined. We attempted to reveal the regulatory mechanism of theanine metabolism through integrated analyses of biochemicals, mRNAs, proteins, and miRNAs.

## Materials and methods

### Experimental design and statistical rationale

Two-year-old cuttings of the tea plant *Camellia sinensis* (L.) O. Kuntze cv. “Longjing 43” were cultivated in an artificial climate chamber at Nanjing Agricultural University. The relative humidity and air temperature were maintained at 70% and 25/18 °C in the daytime/nighttime, respectively. The daytime/nighttime period was 14/10 h, and the light intensity was 300 μmol m^−2^ s^−1^. The plant materials were grown in half-strength nutrient solution before treatment. Then, control tea seedlings were transplanted to full-strength nutrient solution, and seedlings intended for N deficiency treatment were transplanted to N-free nutrient solution. The composition of the full-strength nutrient solution was as described by Wan et al.^47^, and the N-free nutrient solution did not include NO^3−^ or NH^4+^. The pH values in all nutrient solutions were controlled at 5.0. The nutrient solution was circulated by pumps for 24 h every day and replaced every 3 days.

To reduce deviation caused by nutrient solution changes, sampling was performed at 3-day intervals when the nutrient solution was replaced. Tea plant shoots (1st, 2nd, and 3rd leaves), old leaves, and lateral roots were harvested after 0, 3, 6 and 9 days of treatment, respectively. The samples were immediately frozen in liquid nitrogen and stored at −80 °C. Each sample was prepared for three independent biological experiments. The samples treated for 0 days were named N^0^S, N^0^O and N^0^R; those treated for 3 days were named N^3+^S, N^3−^S, N^3+^O, N^3−^O, N^3+^R, and N^3−^R; those treated for 6 days were named N^6+^S, N^6−^S, N^6+^O, N^6−^O, N^6+^R, and N^6−^R; and those treated for 9 days were named N^9+^S, N^9−^S, N^9+^O, N^9−^O, N^9+^R, and N^9−^R.

### Determination of enzyme activity and amino acid content

The samples of tea plant shoots, old leaves, and roots were weighed and then powdered in liquid nitrogen. According to the instructions of a kit produced by KeMing Biotechnology Co., Ltd. (Suzhou, China), the crude enzyme solutions of GS, GOGAT, GDH, NR, and ALT were extracted. The optical density (OD) values were determined with the microplate method using a microplate reader at 540, 340, 340, 540, and 505 nm. Then, the activity of the enzymes was calculated based on the fresh weight (FW) using the corresponding calculation formulas.

The powdered samples were dried for 3 days in a freeze dryer. Theanine was extracted and quantified according to our reported method using ultra-performance liquid chromatography^42^. The other 16 free amino acids were extracted according to the method of Zhao et al.^48^ and then analyzed by an automatic amino acid analyzer (Hitachi L-8900, Tokyo, Japan), including glutamate (Glu), alanine (Ala), arginine (Arg), valine (Val), leucine (Leu), aspartate (Asp), threonine (Thr), lysine (Lys), methionine (Met), isoleucine (Ile), serine (Ser), glycine (Gly), cysteine (Cys), histidine (His), tyrosine (Tyr), and phenylalanine (Phe).

### Transcriptome sequencing and analysis

Tea plant shoots and roots under the control and nitrogen deficiency treatments for 6 days were selected for sequencing (N^6+^S, N^6−^S, N^6+^R, and N^6−^R). Transcriptome sequencing and data analysis in reference to the tea tree genome (http://www.plantkingdomgdb.com/tea_tree/) were accomplished by BGI Gene Tech Co., Ltd. (Shenzhen, China) on a BGISEQ-500 platform. The filtration of raw reads and alignment of transcripts to the reference genome were conducted with the software SOAPnuke v1.5.2 (https://github.com/BGI-flexlab/SOAPnuke) and Hierarchical Indexing for Spliced Alignment of Transcripts (HISAT) v2.0.4 (http://www.ccb.jhu.edu/software/hisat), respectively. Then, the software programs StringTie v1.0.4 (http://ccb.jhu.edu/software/stringtie), Cufflinks v2.2.1 (http://cole-trapnell-lab.github.io/cufflinks) and CPC v0.9-r2 (http://cpc.cbi.pku.edu.cn) were successively used to predict new transcripts. Following statistical analysis of the gene alignment ratio with Bowtie2 v2.2.5 (http://bowtie-bio.sourceforge.net/Bowtie2/index.shtml), the gene expression levels were calculated by RSEM v1.2.12 (http://deweylab.biostat.wisc.edu/RSEM). Based on the PossionDis, the DEGs identified with criteria of an *FDR* (false discovery rate) ≤0.001 and a fold change ≥2.00 were filtered from the four group comparisons, including N^6−^R/N^6+^R, N^6−^S/N^6+^S, N^6+^R/N^6+^S, and N^6−^R/N^6−^S.

### Proteome sequencing and analysis

Isobaric tags for relative and absolute quantitation (iTRAQ) technology and a Triple TOF 5600 mass spectrometer (MS) (AB SCIEX, Concord, ON, Canada) were used for protein quantification and qualification. Proteins were successively extracted, alkylated, quantified by Bradford assays, analyzed for expression by sodium dodecyl sulfate polyacrylamide gel electrophoresis (SDS-PAGE), and trypsin digested. Then, the four samples were labeled with iTRAQ tags 114 (N^6−^S), 116 (N^6+^S), 117 (N^6−^R), and 119 (N^6+^R). Each labeled sample was pooled and separated by an LC-20AB liquid chromatography (LC) system (Shimadzu, Japan) with an Ultremex strong cation exchange (SCX) column (4.6 × 250 mm). The LC fractions were analyzed on a Triple TOF 5600 MS. Based on Mascot 2.3.02 software, the proteins were identified from the converted MS raw data against the UniProt plant database (https://www.ncbi.nlm.nih.gov). According to criteria of a fold change > 1.2 and a *P* value < 0.05, the DEPs were identified for the N^6−^R/N^6+^R, N^6−^S/N^6+^S, N^6+^R/N^6+^S, and N^6−^R/N^6−^S comparisons.

### MicroRNA sequencing and analysis

Total RNA was first isolated from samples, and then small RNAs (18–30 nucleotides) were recovered by PAGE and subsequently ligated with 3′ and 5′ adaptors. A small-RNA library was established after reverse transcription, PCR amplification and purification of the RNA fragments with adaptors. An Agilent 2100 Bioanalyzer and an ABI StepOnePlus Real-Time PCR System were used for quality and quantity detection, respectively, and an Illumina HiSeq 2000 sequencing system was used for sequencing of the generated small-RNA library.

High-quality clean full-length tags were obtained through quality control (QC) and filtration of raw data. The extracted clean reads were mapped to the tea tree genome (http://www.plantkingdomgdb.com/tea_tree/) using Bowtie2. Then, the clean tags were aligned with known miRNA sequences from miRBase (http://www.mirbase.org/) and matched with rRNAs, tRNAs, snRNAs, snoRNAs, repeats, exons, introns, and other elements. The nonannotated clean tags were used to predict novel miRNAs on the basis of the biological characteristics of miRNAs by MirDeep v0.2 (http://sourceforge.net/projects/mireap/files/latest/download). The DEMs were filtered with criteria of a fold change ≥ 2 and an FDR ≤ 0.01 by DEGseq v1.18.0 and DESeq2 v1.4.5. Then, psRobot v1.2 and TargetFinder v1.0 were employed to predict miRNA target genes.

### Real-time quantitative PCR (RT-qPCR) analysis of mRNA expression

Total RNA was extracted from samples using an RNA Isolation Kit (Aidlab, Beijing, China) and then reversed transcribed into cDNA using a PrimeScript RT Reagent Kit (TaKaRa, Dalian, China). A CFX96 RT-qPCR platform (Bio-Rad, Hercules, CA, USA) was used for RT-qPCR analysis. Each reaction system contained 10 μL of SYBR Premix *Ex Taq* fluorescent reagent (TaKaRa), 7.2 μL of ddH_2_O, 2 μL of diluted cDNA, and 0.4 μL of forward/reverse primer. The RT-qPCR reaction conditions were as follows: 95 °C for 30 s, 40 cycles at 95 °C for 5 s and 60 °C for 30 s, and 61 cycles at 65 °C for 10 s. Each sample was performed in triplicate, and the average and standard deviation were calculated. The gene primers for RT-qPCR are listed in Table S1.

### RT-qPCR analysis of miRNA expression

miRNAs were isolated from samples according to the instructions accompanying an EASYspin plant miRNA isolation kit (Aidlab). Based on the poly (A) tailing principle and the miRNAs were reverse transcribed into cDNA with a miRNA cDNA first-strand synthesis kit (Aidlab). 5S rRNA was used as the internal reference. Then, RT-qPCR was performed with a reaction system of 2 μL of diluted cDNA, 0.4 μL (0.2 μM) of forward/reverse primer, 10.0 μL of 2× SYBR Green Mix (Aidlab), and 7.2 μL of ddH_2_O. The reactions were carried out in triplicate on a CFX96 RT-qPCR system under the following amplification conditions: denaturation at 94 °C for 2 min followed by 40 cycles at 94 °C for 15 s and 60 °C for 40 s. RT-qPCR forward primers were designed for the miRNAs and are listed in Table S2.

### Statistical analyses

Based on the significant DEPs, the matching mRNAs were identified. The expression levels and up-/downregulation of significant DEPs and all DEGs were quantified. Then, the correlated DEGs were further analyzed with Gene Ontology (GO) and Kyoto Encyclopedia of Genes and Genomes (KEGG) pathway functional enrichment analyses. Using ACGT101-CORR 1.1, the correlations between significant DEGs and DEMs were analyzed. The target genes negatively correlated with the miRNAs were further subjected to GO and KEGG analyses.

The cycle threshold (CT) values of miRNAs and the mRNA transcript levels determined from RT-qPCR were used to calculate the expression levels relative to the 5S and *Csβ-actin* genes, respectively, by the 2^−∆∆CT^ method^42^. The mean values and standard deviations (SDs) for the levels of gene expression, the levels of 17 amino acids and the activity of enzymes were calculated with three independent biological replicates. Using SPSS 17.0 software, the correlations between theanine content and gene expression levels and between proteinogenic amino acid content and enzyme activity were analyzed with the Pearson method.

## Results

### Identification of DEPs, DEGs, and DEMs involved in the theanine metabolism pathway

**Fig. 1:**
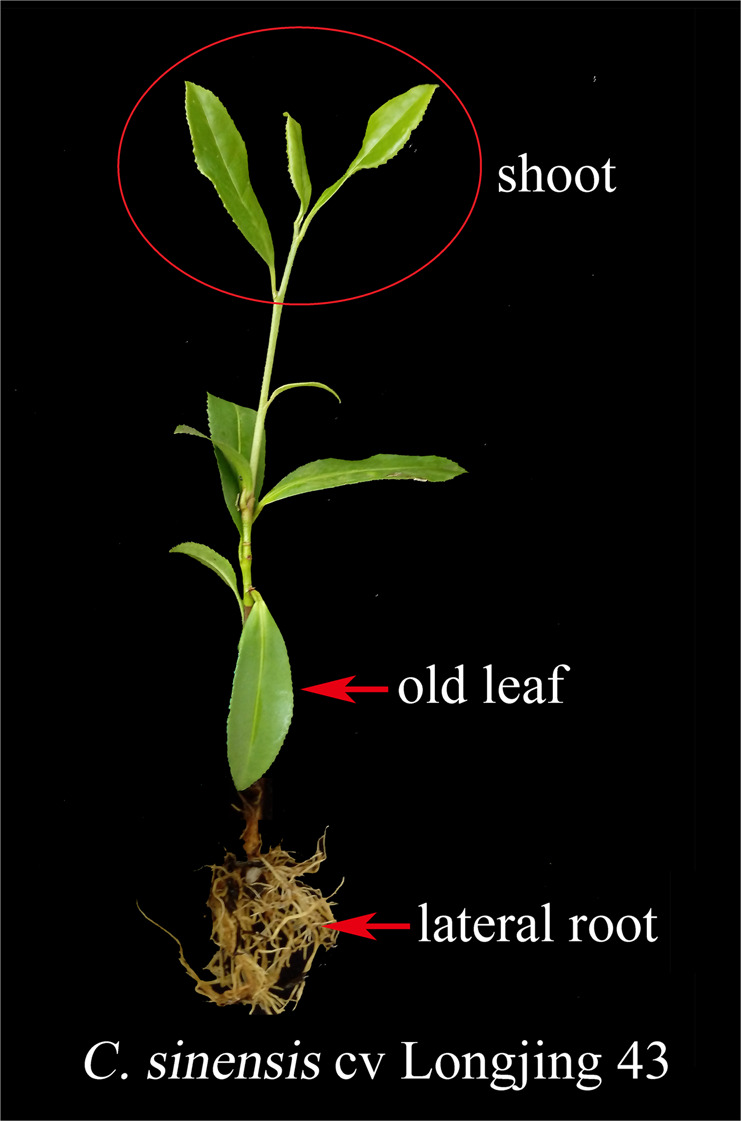
Two-year-old cutting of a tea plant (*Camellia sinensis* cv. “Longjing 43”)

**Fig. 2:**
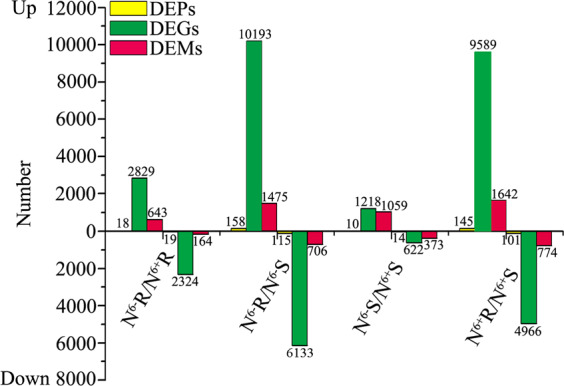
Statistics for the DEGs, DEPs and DEMs in the N^6−^R/N^6+^R, N^6−^S/N^6+^S, N^6+^R/N^6+^S and N^6−^R/N^6−^S groups

Different tissues of *C. sinensis* cv. “Longjing 43” were harvested as samples (Fig. 1). The sampling scheme for the tea plants under different nitrogen treatments is described in Table 1. Four samples, N^6−^R, N^6+^R, N^6−^S, and N^6+^S, were used for RNA-seq, proteome analysis and miRNA sequencing, and the data were analyzed through four comparisons, N^6−^R/N^6+^R, N^6−^S/N^6+^S, N^6+^R/N^6+^S, and N^6−^R/N^6−^S. From the four samples, 38,998 genes were obtained after comparison with the tea plant genome, including 28,812 known genes and 10,186 novel genes. A total of 4912 proteins and 6028 known miRNAs were identified after alignment with the UniProt plant database and plant miRbase, respectively. Moreover, 2829 novel miRNAs were predicted, including 282 in N^6−^R, 297 in N^6+^R, 991 in N^6−^S, and 1259 in N^6+^S. The target genes of all miRNAs and the differentially expressed miRNAs were predicted, and the known and novel miRNA−mRNA pairs were counted (Table S3). All the DEPs, DEGs, and DEMs were counted; in all comparison groups, the numbers of DEPs were the lowest, while the numbers of DEGs were the highest. Among the four groups, the numbers of DEPs, DEGs and DEMs in the comparisons of the same tissues under different N conditions were significantly lower than those in the comparisons of different tissues under the same N conditions (Fig. 2).

**Table 1:** Sampling of tea plants under different nitrogen treatments

Treatment	Control	Nitrogen sufficiency			Nitrogen deficiency		
Time	0 days	3 days	6 days	9 days	3 days	6 days	9 days
Shoot	N^0^S	N^3+^S	N^6+^S	N^9+^S	N^3−^S	N^6−^S	N^9−^S
Old leaf	N^0^O	N^3+^O	N^6+^O	N^9+^O	N^3−^O	N^6−^O	N^9−^O
Root	N^0^R	N^3+^R	N^6+^R	N^9+^R	N^3−^R	N^6−^R	N^9−^R

*S* shoot, *O* old leaf, *R* root, + full-nutrient treatment, − nitrogen deficiency treatment

**Fig. 3:**
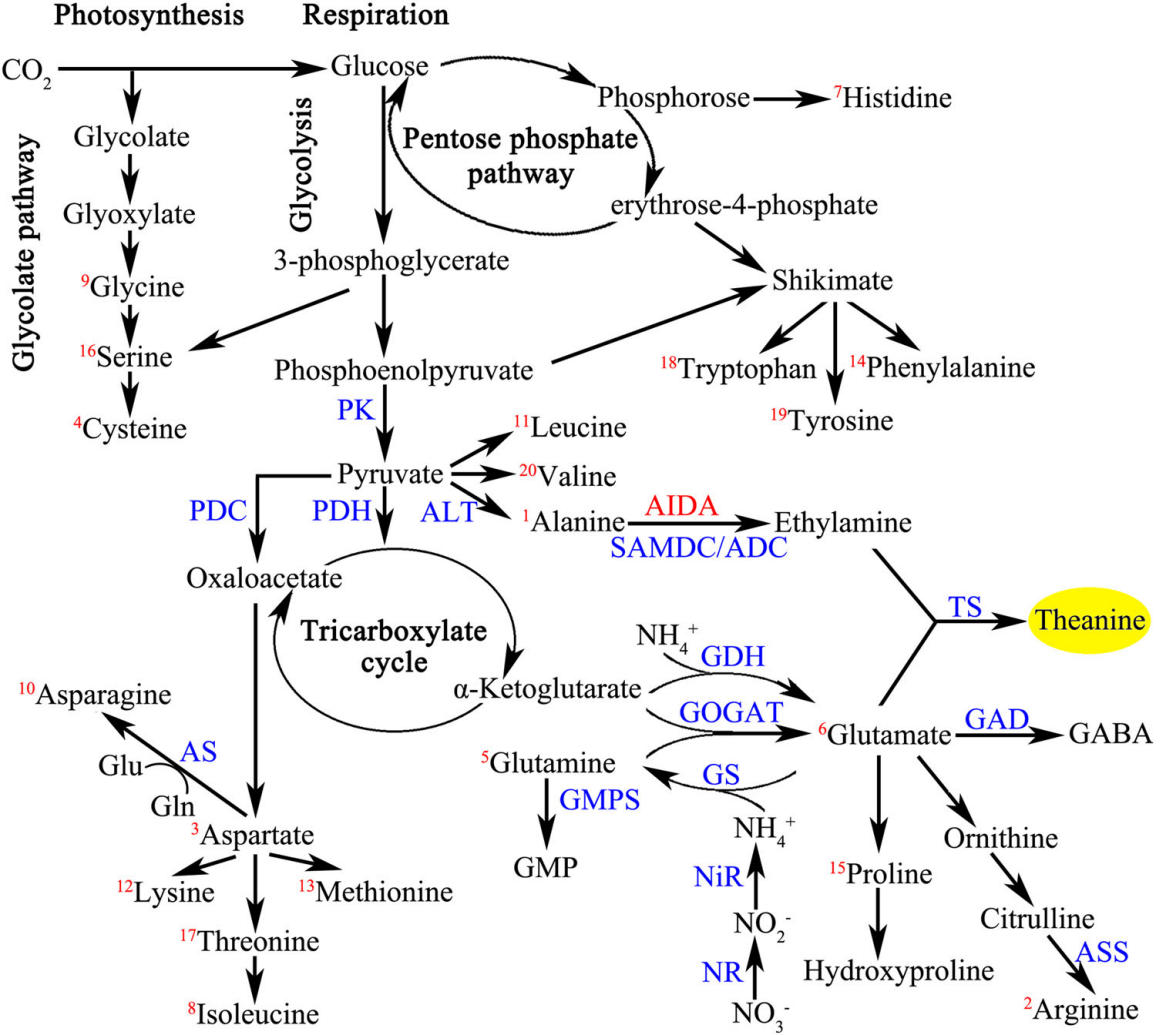
Schematic of 20 free proteinogenic amino acids and the theanine biosynthesis pathway in the tea plant. Note: The 20 free proteinogenic amino acids are marked with red numbers in the top left corner

**Fig. 4:**
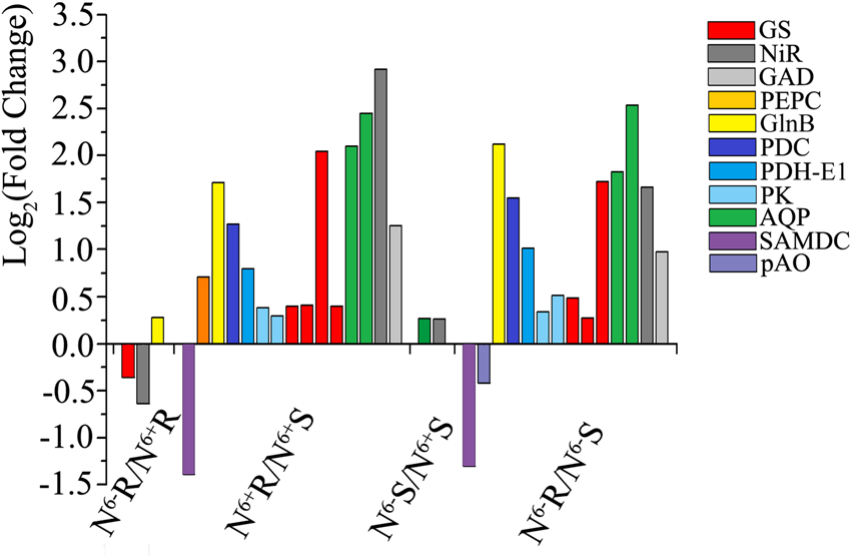
Expression profiles of the DEPs involved in theanine metabolism

Based on a schematic of the theanine biosynthesis pathway in the tea plant including 20 proteinogenic amino acids and one unique nonproteinogenic amino acid (Fig. 3), the DEPs related to theanine metabolism were identified. Within the same tissues, only one upregulated DEP, GlnB, and two downregulated DEPs, GS and NiR, were identified in N^6−^R/N^6+^R, while two upregulated DEPs, AQP and NiR, were identified in N^6−^S/N^6+^S. In both the N^6+^R/N^6+^S and N^6−^R/N^6−^S comparisons, ten kinds of DEPs were detected, nine of which showed similar expression profiles. Only one upregulated DEP, PEPC, and one downregulated DEP, pAO, were specific to N^6+^R/N^6+^S and N^6−^R/N^6−^S, respectively (Fig. 4).

The differentially expressed genes of 24 proteins or enzymes participating in N and theanine metabolism were investigated according to their fragments per kilobase of exon model per million mapped fragments (FPKM) values. In addition, the identified corresponding miRNAs of those DEGs are listed in Table S4. Several DEGs were detected only when shoots and roots were compared, including *GOGAT*, *GAD*, *GMPS*, *ALT*, *ADC*, *GlnB*, *PDC*, *PDH-E1*, and *PDH-E2*. In all four comparisons, the total levels of the DEGs *NRT*, *GLR*, *AMT*, *AQP*, and *PDP* were obviously higher than those of the other genes. Moreover, there were more upregulated filtered DEGs than downregulated genes (Table S5). DEGs belonging to the same family may show different expression profiles. The expression levels of selected DEGs involved in theanine metabolism were verified by RT-qPCR, and the up-/downregulation profiles were consistent with transcriptome predictions (Table S6).

### Correlation analyses of the transcriptome and proteome data

There were 4700 overlapping molecules between all 4912 expressed proteins from the proteome and all 38,998 expressed transcripts from the transcriptome. Then, the expression levels and up-/downregulation correlations between significant DEPs and all DEGs were analyzed (Table S7). There were 8, 1, 157, and 138 all DEP−DEG correlation pairs in the N^6−^R/N^6+^R, N^6−^S/N^6+^S, N^6−^R/N^6−^S, and N^6+^R/N^6+^S comparisons, respectively. There were more DEP–DEG pairs in the comparisons between shoots and roots under the same N conditions than in the comparisons between different N conditions for the same tissues, and there were more positive correlations (protein upregulation with transcript upregulation (P-Up_T-Up)/protein downregulation with transcript downregulation (P-Down_T-Down) than negative correlations (protein upregulation with transcript downregulation (P-Up_T-Down)/protein downregulation with transcript upregulation (P-Down_T-Up).

In N^6−^R/N^6−^S, several theanine metabolism-related proteins showed P-Down_T-Down (*SAMDC*, CSA034593), P-Up_T-Down (*GlnB*, CSA009523), and P-Up_T-Up (*NiR*, CSA003866; *PDH-E1*, CSA007666). In N^6+^R/N^6+^S, the proteins *PDH-E1* (CSA004843) exhibited P-Up_T-Down, while *NiR* (CSA003866), *PDH-E1* (CSA007666), *AQP* (CSA000157), *GS* (CSA030118), and *PDC* (CSA002466) exhibited P-Up_T-Up (Table 2). In N^6−^R/N^6+^R and N^6−^S/N^6+^S, no corresponding pairs of theanine biosynthesis-related proteins and mRNAs were found.

**Table 2:** Information of DEP−DEG correlation pairs involved in theanine metabolism

Group	Gene ID	log_2_Fold (DEGs)	log_2_Fold (DEPs)	Gene name
N^6−^R/N^6−^S	CSA034593	−1.994938474	−1.300448367	*SAMDC*
	CSA009523	−1.129114488	2.120020096	*GlnB*
	CSA003866	1.992394584	1.6617496	*NiR*
	CSA007666	1.763353188	0.571434116	*PDH-E1*
N^6+^R/N^6+^S	CSA000157	7.703267978	2.099968417	*AQP*
	CSA003866	1.154804323	2.918195389	*NiR*
	CSA007666	1.884338053	0.60312187	*PDH-E1*
	CSA004843	−1.530292302	0.799087306	*PDH-E1*
	CSA030118	5.099742657	2.045093438	*GS*
	CSA002466	1.227809566	0.611644543	*PDC*

**Fig. 5:**
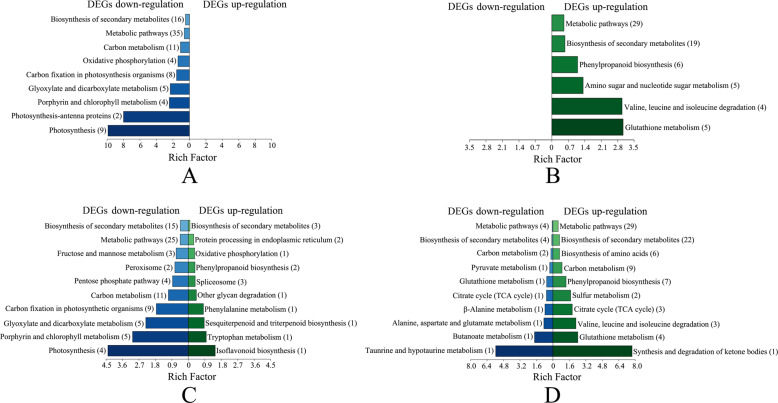
KEGG analyses of DEGs correlated with DEPs. **a** DEPs downregulated in N^6−^R/N^6−^S; **b** DEPs upregulated in N^6−^R/N^6−^S; **c** DEPs downregulated in N^6+^R/N^6+^S; **d** DEPs upregulated in N^6+^R/N^6+^S

The DEGs correlated with DEPs in N^6−^R/N^6−^S and N^6+^R/N^6+^S were further classified through KEGG pathway analyses. In N^6−^R/N^6−^S, only DEGs with expression positively correlated with DEP expression were enriched for pathway terms. The term “Biosynthesis of secondary metabolites”, followed by the term “Metabolic pathways”, had the greatest number of enriched genes. In addition, the range of enrichment factors (EFs) was wider for downregulated proteins (0−10) than for upregulated proteins (0−3.5), while the opposite trend was observed for N^6+^R/N^6+^S. In N^6+^R/N^6+^S, the DEGs of the P-Up_T-Down pairs were enriched for the terms “Pyruvate metabolism”, “TCA cycle”, “β-Alanine metabolism”, and “Alanine, aspartate and glutamate metabolism”, and the DEGs of the P-Up_T-Up pairs were enriched for the terms “Biosynthesis of amino acids” and “Valine, leucine, and isoleucine degradation” (Fig. 5). All these pathway terms are closely related to theanine metabolism in the tea plant.

### Correlation analyses of mRNAs and microRNAs

**Fig. 6:**
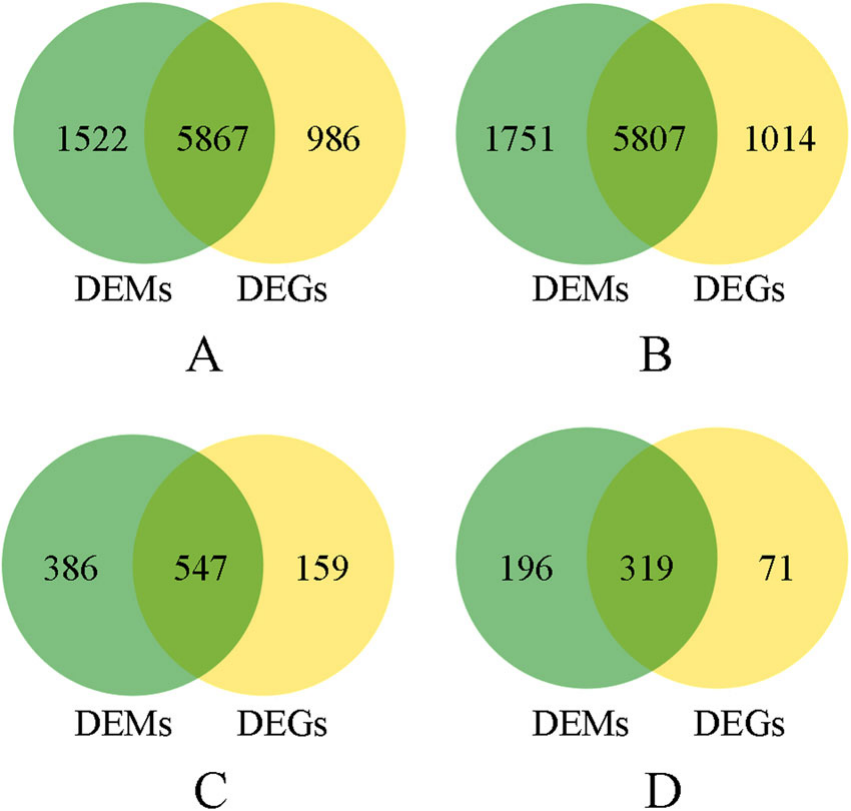
Numerical statistics for the negative miRNA−mRNA interactions. **a** N^6−^R/N^6−^S; **b** N^6+^R/N^6+^S; **c** N^6−^R/N^6+^R; **d** N^6−^S/N^6+^S

The significant DEMs and their predicted targets were investigated for cognate mRNA targets to determine miRNA−mRNA functional interactions. There were 5867, 5807, 547 and 319 negative DEM−DEG interactions in N^6−^R/N^6−^S, N^6+^R/N^6+^S, N^6−^R/N^6+^R, and N^6−^S/N^6+^S, respectively (Fig. 6). Among them, five interactions in N^6−^R/N^6−^S involved the theanine biosynthesis-related genes *GOGAT*, *GLR*, and *PDH-E2*; six interactions in N^6+^R/N^6+^S involved the theanine biosynthesis-related genes *GOGAT*, *GLR*, *NiR*, *SAMDC*, and *PDP*; one interaction in N^6−^R/N^6+^R involved the theanine biosynthesis-related gene *GLR*; and two interactions in N^6−^S/N^6+^S involved the theanine biosynthesis-related genes *PK* and *PDP*. These genes corresponded to nine miRNAs, which negatively interacted with 59 mRNAs in all four comparisons (Fig. S1). Using RT-qPCR, the up-/downregulation of DEMs and DEGs in negative DEM−DEG interactions related to theanine metabolism was verified. The validation results for regulation were consistent with the sequencing results in Table S8.

The DEGs negatively interacting with DEMs were further subjected to GO and KEGG pathway enrichment analyses. Among the 30 most enriched GO terms for N^6−^R/N^6−^S, the significant terms were “integral component of membrane”, “plasma membrane” and “plasmodesma” in the cellular component (CC) category and “defense response” in the biological process (BP) category. For N^6+^R/N^6+^S, the significant terms were “plasma membrane” in the CC category and “defense response” in the BP category. For N^6−^R/N^6+^R, the significant terms were “microtubules” and “kinesin complexes” in the CC category (Fig. S2).

**Fig. 7:**
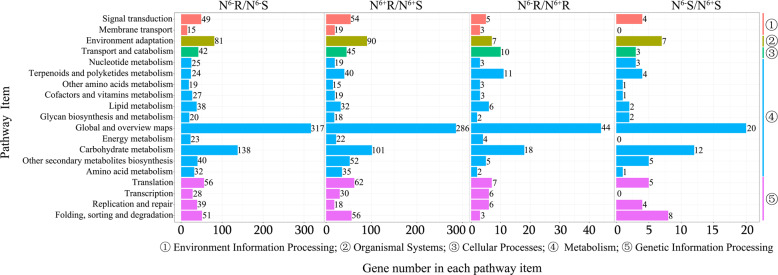
KEGG analysis results for the DEGs negatively correlated with DEPs

In the KEGG classification system, the DEGs were classified into five biochemical pathway categories (level 2), including the environmental information processing (2 items), organismal system (1 pathway), cellular processes (1 pathway), metabolism (11 pathways), and genetic information processing (4 pathways) categories (Fig. 7). The metabolism category had the most pathways and genes. There were 51, 50, 5 and 2 genes enriched for amino acid metabolism in N^6−^R/N^6−^S, N^6+^R/N^6+^S, N^6−^R/N^6+^R, and N^6−^S/N^6+^S, respectively.

### Dynamic changes in the content of 17 amino acids

The dynamic changes in the content of 17 free amino acids in tea plants are shown in Table S9. In general, the total amounts of amino acids in roots and shoots were far greater than those in old leaves. As time passed, the amino acid content increased, but the increments in N^+^ tissues were greater than those in N^−^ tissues. In addition, the increments from the third day to the sixth day were significant, but the changes between the sixth day and ninth day were not obvious. Theanine, glutamate, arginine, aspartate, serine, and phenylalanine made up the majority of the total amino acid content. Among the 17 amino acids, theanine had the highest content in roots, while glutamate had the highest content in both shoots and old leaves.

The theanine content in old leaves was the lowest, while that in roots was the highest. In N^+^S, N^−^S and N^+^R, theanine content was slightly decreased on the third day, significantly increased on the sixth day and slightly decreased on the ninth day. In contrast, the theanine content in roots gradually increased when tea plants were under N deficiency. On the third day, the theanine levels in N^+^S and N^+^R were lower than those in N^−^S and N^−^R. However, the theanine levels in N^6+^S, N^6+^O, N^6+^R, N^9+^S, and N^9+^O were significantly higher than those in the same tissues under N-free conditions, while the theanine content in N^9+^R was close to that in N^9−^R.

### Correlation analyses of the content of theanine and the expression levels of genes involved in theanine metabolism

**Fig. 8:**
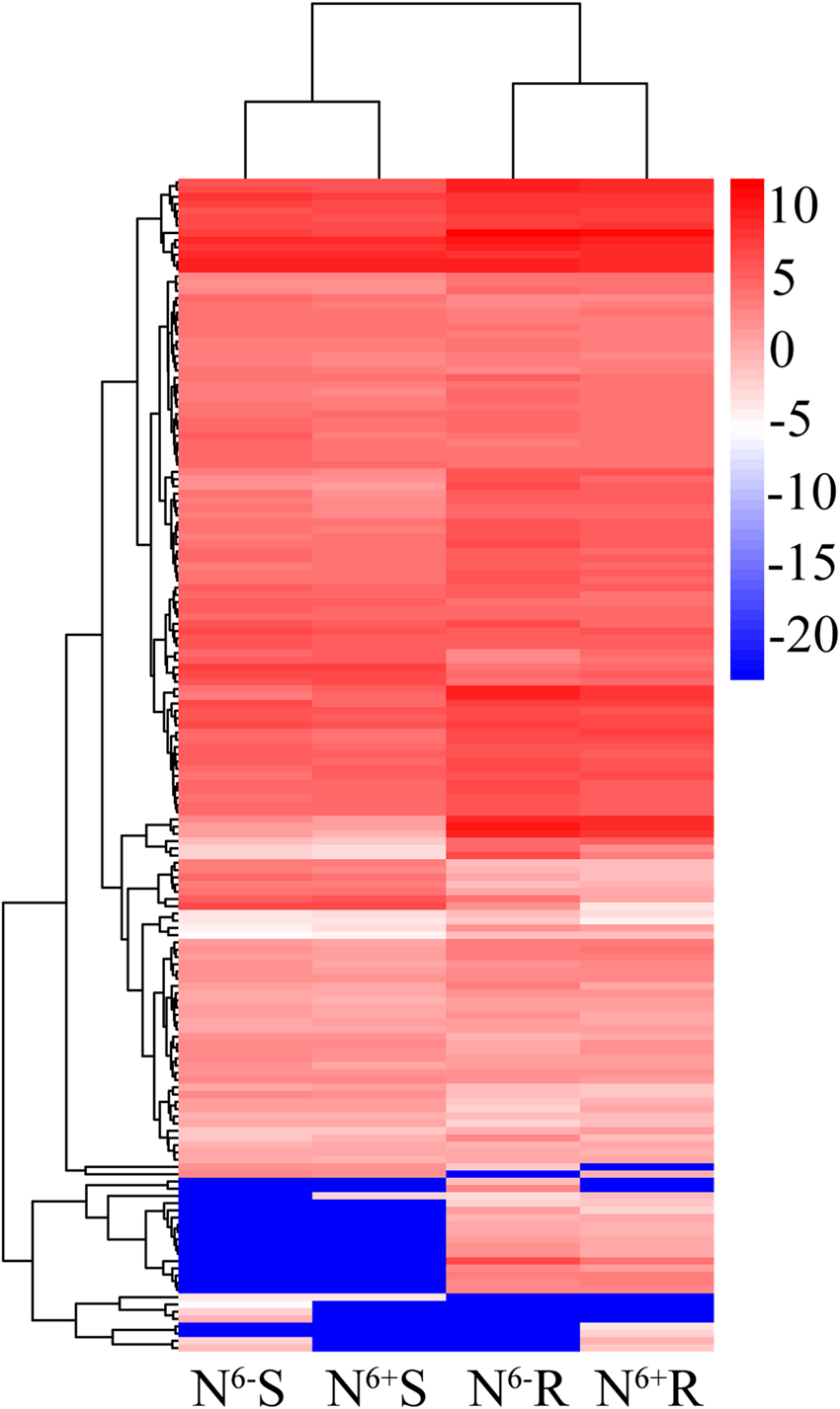
Cluster analyses of identified genes involved in theanine metabolism

We identified the expression of all genes involved in theanine metabolism from the transcriptome, including 1 *NR*, 1 *NiR*, 4 *GS*, 4 *GOGAT*, 3 *GDH*, 3 *ALT*, 2 *ADC*, 3 *SAMDC*, 4 *NRT*, 4 *GAD*, 4 *AS*, 2 *GMPS*, 12 *GLR*, 7 *AO*, 10 *PEPC*, 11 *PK*, 2 *ASS*, 13 *AMT*, 1 *GlnB*, 8 *PDC*, 15 *PDP*, 2 *PDK*, 15 *PDH*, and 31 *AQP* genes. Cluster analysis revealed that most of these 162 genes showed higher expression levels in roots than in shoots (Fig. 8; Table S10). However, the genes showed different expression profiles even though they belonged to the same families. For example, the transcript levels of *GSb* in roots were higher than those in shoots, but the results for *GSa* and *GSc* were opposite. *NADH-GOGATb* and *NADH-GOGATc* exhibited similar profiles that differed from those of *NADH-GOGATa*. All three *NADH-GOGAT* genes were expressed at higher levels in N^6+^R than in N^6−^R, while they were expressed at lower levels in N^6+^S than in N^6−^S.

The correlation analyses indicated that a certain degree of correlation existed between theanine content and related gene expression levels (Table S11). The *NiR*, *NADH-GOGATa*, *NRT2.5b*, *AMT3-1d*, *AMTa*, *GADc*, and *AQP19a* levels were significantly positively correlated with theanine levels, while the *GLR3.7a*, *PEPC4a*, *PEPC4b*, *AMT3.1b*, and *PDH-E1α4* levels showed significant negative correlations. All of the genes of the *GDH*, *ADC*, *SAMDC*, *NRT*, *GAD*, and *PDK* families showed positive correlations with theanine content. In addition, the *NR*, *Fd-GOGAT*, *ASS*, and *GlnB* levels were negatively correlated with theanine content. However, different genes belonging to one family showed different correlations with theanine content, such as *GS*, *GOGAT ALT*, *AS*, *GMPS*, *GLR*, *AO*, *PEPC*, *PK*, *AMT*, *PDC*, *PDP*, *PDH*, and *AQP*.

### Activity of five structural enzymes

**Fig. 9:**
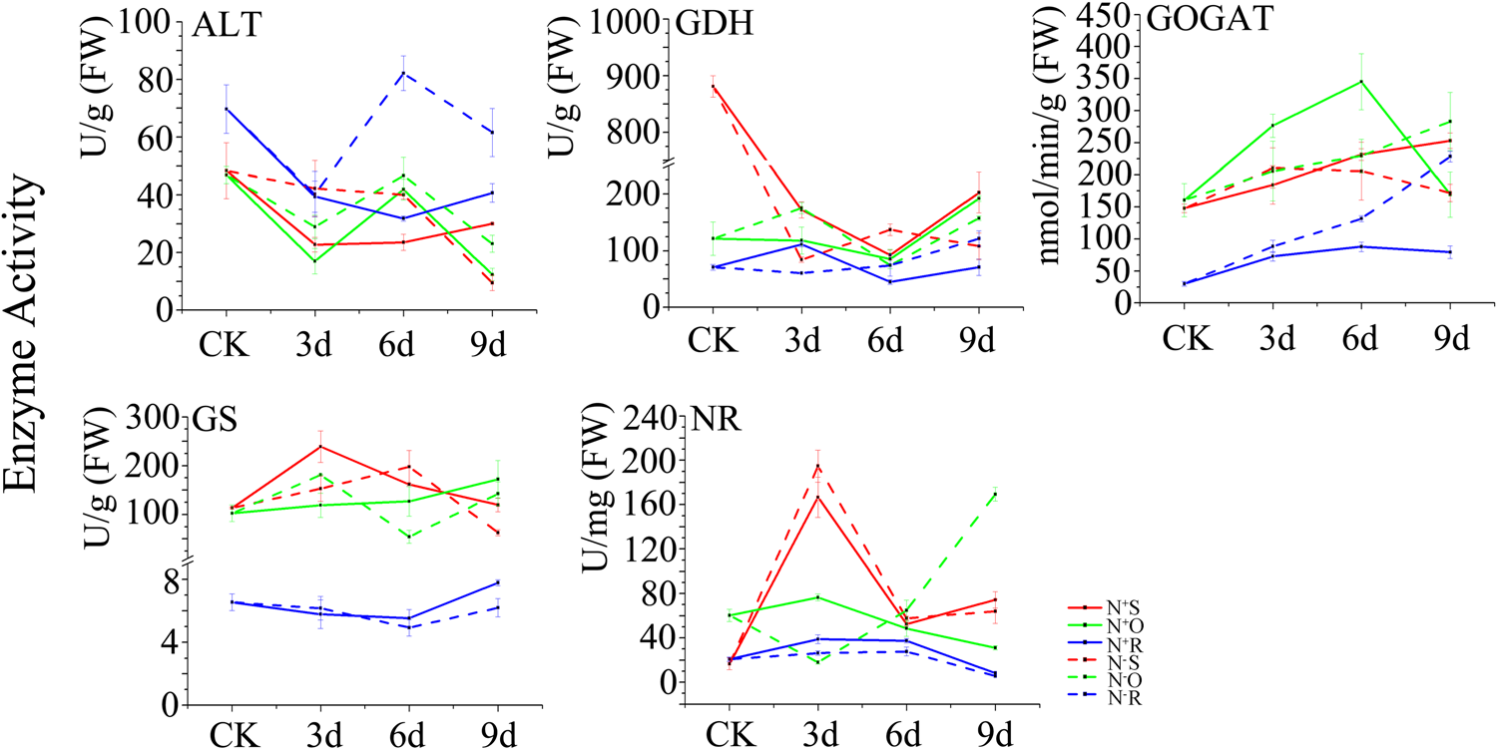
Dynamic changes in the activity of enzymes involved in theanine metabolism

We selected five structural enzymes involved in the theanine biosynthesis pathway, including ALT, GDH, GOGAT, GS, and NR, and detected their activity (Fig. 9). Among different tissues, the ALT activity between N^+^S and N^+^R, the ALT and GOGAT activity between N^−^O and N^−^R, and the GDH activity between N^+^S and N^+^O were similar. Among the same tissues, the ALT activity between N^+^O and N^−^O, the GS and NR activity between N^+^R and N^−^R, and the NR activity between N^+^S and N^−^S showed similar dynamic changes.

### Correlation analyses between theanine levels and related amino acid levels and enzyme activity

**Fig. 10:**
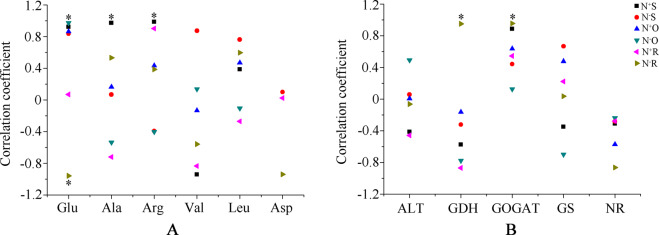
Correlation analyses between the content of theanine and the levels of six free proteinogenic amino acids (**a**) and the activity of five related enzymes (**b**). * indicates significance at *P* < 0.05

The correlations between the levels of theanine and those of six related proteinogenic amino acids were analyzed, including Glu, Ala, Arg, Val, Leu, and Asp (Fig. 10a). Among different tissues, there was a strong correlation in N^6+^S between the levels of theanine and those of amino acids except Leu. In N^6+^S, the theanine content showed a significant correlation with the Ala and Arg content. Among the six amino acids, the Glu was strongly correlated with theanine, while Leu was weakly correlated with theanine, in most instances with regard to content. The levels of Glu and theanine were significantly correlated in N^−^O and N^−^R.

The correlations between theanine content and enzyme activity were analyzed (Fig. 10b). GOGAT and NR activity showed positive and negative correlations, respectively, with theanine content in all tissues. GDH activity was negatively correlated with theanine content, except for a significant positive correlation in N^−^R. Moreover, GOGAT activity was also significantly positively correlated with theanine content in N^−^R.

## Discussion

### DEPs, DEGs, and DEMs involved in theanine metabolism

From the results, the numbers of DEGs in the four comparisons were significantly higher than those of DEPs. In particular, the difference multiples for the DEG and DEP numbers in N^6−^R/N^6+^R and N^6−^S/N^6+^S were greater than those in N^6−^R/N^6−^S and N^6+^R/N^6+^S. This result indicated that genes were more sensitive to external environmental factors than proteins. As previous reports have indicated, multistep reactions and regulation are needed during the progression from gene expression to protein expression^49^. Theanine content in both tissues was higher in N^+^ samples than in N^−^ samples. The DEGs, DEPs and DEMs showed more significant differences between different tissues than between different N levels. N may regulate theanine synthesis not only by regulating the expression of genes, proteins and miRNAs but also by influencing other physiological and biochemical processes.

In N^6−^R/N^6+^R and N^6−^S/N^6+^S, only three and two DEPs were identified, respectively, and showed different expression profiles. The results revealed that the influence of N deficiency on protein expression was different in different tissues due to tissue specificity. In N^6−^R/N^6−^S and N^6+^R/N^6+^S, the kinds and expression profiles of the DEPs were similar. The differences in protein expression between shoots and roots were slightly affected by N availability. However, most proteins related to theanine metabolism, such as GlnB, PDC1, PDK, PDH-E1, GS, AQP, NiR, and GAD, were upregulated in roots compared with shoots. *PDC1* is predominantly present in the roots of *Arabidopsis*^50^. PIP-type *AQP* gene expression is higher in the roots than in the leaves and stems of *Galega orientalis*^51^. The synthetic pathway of theanine shows that high expression of NiR, GS, and PDH-E1 contributes to glutamate generation, while downregulation of GlnB has been demonstrated to be beneficial for nitrite uptake and amino acid accumulation^52^. In addition, PEPC upregulation in N^6+^R/N^6+^S and pAO downregulation in N^6−^R/N^6−^S both contributed to theanine accumulation. We speculate that the higher theanine content in roots than in shoots was most likely related to protein expression profiles.

Among the DEGs related to theanine metabolism, the transportation-related genes *NRT*, *GLR*, *AMT* and *AQP* were present at obviously higher levels than other genes. Furthermore, most of these genes showed upregulation, while some structural genes of theanine synthesis showed no differential expression in N^6−^R/N^6+^R and N^6−^S/N^6+^S. These results indicated that several genes are key to regulating ammonium uptake and assimilation^15^. In this study, we found that *NRT2.5b*, *AMT3-1d*, *AMTa*, and *AQP19a* levels showed significant positive correlations with theanine content. In addition, the expression of these genes was influenced in roots when N availability changed. Theanine synthesis may be indirectly affected by N uptake and transport under different N availability conditions.

### Correlation analyses of DEPs, DEGs, and DEMs

The identified DEP−DEG correlation pairs mostly belonged to N^6−^R/N^6−^S and N^6+^R/N^6+^S. Only the DEGs positively correlated with proteins in N^6−^R/N^6−^S were enriched in KEGG pathways. Most of those genes were enriched for the “Biosynthesis of secondary metabolites” and “Metabolic pathways” terms. The results revealed that metabolism was regulated by genes and corresponding proteins with the same expression trends in tea plants under N deficiency. The upregulated DEPs in N^6+^R/N^6+^S were enriched for amino acid metabolism terms closely related to theanine biosynthesis. It has been shown that amino acid metabolism is stronger under N sufficiency than under N deficiency^15^, which is consistent with the elevated theanine levels in tea plants under N sufficiency at the molecular level.

In both N^6−^R/N^6−^S and N^6+^R/N^6+^S, the DEGs negatively correlated with DEMs were enriched for the term “plasma membrane” in the CC category and the term “defense response” in the BP category. The plasma membrane is a site of cell material exchange and information transmission, and defense responses are related to nutritional conditions for tea roots^53,54^. In addition, most of the DEGs were enriched for the “metabolism” term. This demonstrated that the metabolic difference between roots and shoots was also regulated by the collective effects of mRNAs and miRNAs.

Negative DEG–DEM interactions involved in theanine metabolism were identified, and the DEGs included the structural genes *GOGAT*, *SAMDC*, *NiR*, *PK*, and *PDH-E2* and the regulatory genes *PDP* and *GLR*. Furthermore, the DEG−DEM interactions were not one-to-one^45^. This shows that the regulatory system of mRNAs and miRNAs in theanine biosynthesis is a complex network. In our study, the identified miRNAs negatively correlated with theanine metabolic genes were mostly novel miRNAs; however, the known miRNA gma-miR156aa interacted with *GLR* and *PK*, and the known miRNA nta-miR156i interacted with *GLR*. Studies have shown that gma-miR156aa is associated with resistance to defoliating insects and fungal pathogens, and nta-miR156i is related to water and chromium stress responses in tobacco roots^55–58^.

### Correlation analyses between the content of theanine and the expression levels of related genes, the content of six proteinogenic amino acids and the activity of five enzymes

Compared with those in shoots, the expression levels of identified genes involved in theanine metabolism in roots were higher with increased theanine content. The number of genes positively correlated with theanine content was greater than the number of genes negatively correlated with theanine content. The critical role of gene expression in theanine accumulation was again verified, supporting our previous research results^13,42^. However, *Fd-GOGAT* and *NADH-GOGAT* show positive and negative correlations, respectively, with theanine content in tea leaves in vitro^42^. In this study, the opposite was true for *Fd-GOGAT* and *NADH-GOGATa*, showing that these genes play different roles in theanine accumulation in tea leaves under different conditions. Different gene families and even different genes belonging to the same family showed different correlations with theanine content, and theanine metabolism was regulated by multiple gene regulation systems.

The maximum and minimum theanine levels were in roots and in old leaves, respectively. It has been confirmed that theanine biosynthesis occurs mainly in roots, and theanine content in leaves gradually decreases as leaf maturity increases^13^. In shoots under N sufficiency, the levels of theanine were strongly correlated with those of Glu, Ala, Arg, Val and Asp, which are closely related to precursors of theanine. It is inferred that theanine synthesis occurs not only in roots but also in shoots, consistent with the findings of previous studies^6,42,59^.

With the dynamic changes in theanine content, the activity of enzymes involved in theanine synthesis also constantly changed. However, the enzyme activity profiles were different in different tissues and under different N levels in most cases. The correlation of theanine content and GOGAT activity was always positive and was the largest in shoots under N sufficiency, while it was the largest in roots under N deficiency. This result indicates that enzyme activity can be influenced by nitrogen treatment and is related to tissue specificity^60,61^. Under N deficiency, the significant positive correlation between GDH and GOGAT activity and theanine content showed that GDH activity was increased under low glutamate concentrations and was used for glutamate synthesis in a supplementary pathway of the GS/GOGAT cycle^62^.

In conclusion, we used, for the first time, an omics strategy to comprehensively analyze the molecular mechanisms of theanine biosynthesis between tea shoots and roots under N sufficiency and deficiency conditions. In addition, we combined biochemical methods to explore the influencing factors related to theanine metabolism. The results showed that theanine biosynthesis was regulated collaboratively by related genes, miRNAs and proteins at the molecular level and was also interrelated with many other factors, such as sites of synthesis sites, the activity of enzymes, and the levels of other related amino acids. In this study, we mainly focused on the influence of N deficiency on theanine synthesis at the molecular level. Due to limitations in the experimental conditions for the tea plants, only two N levels (full N and zero N) were used as treatments. The comprehensive regulatory mechanism of N for theanine synthesis needs more in-depth and detailed research. Therefore, it is necessary to set up N concentration gradients for treatments in future research.

## Supplementary information

Figure S1

Figure S2

Table S1–11
