# Supplementary figures and images for: Integrative transcriptome, proteome, and microRNA analysis reveals the effects of nitrogen sufficiency and deficiency conditions on theanine metabolism in the tea plant (Camellia sinensis)
Source: Hortic Res. 2020 May 1;7:65. doi: 10.1038/s41438-020-0290-8 (PMC7192918; doi:10.1038/s41438-020-0290-8)

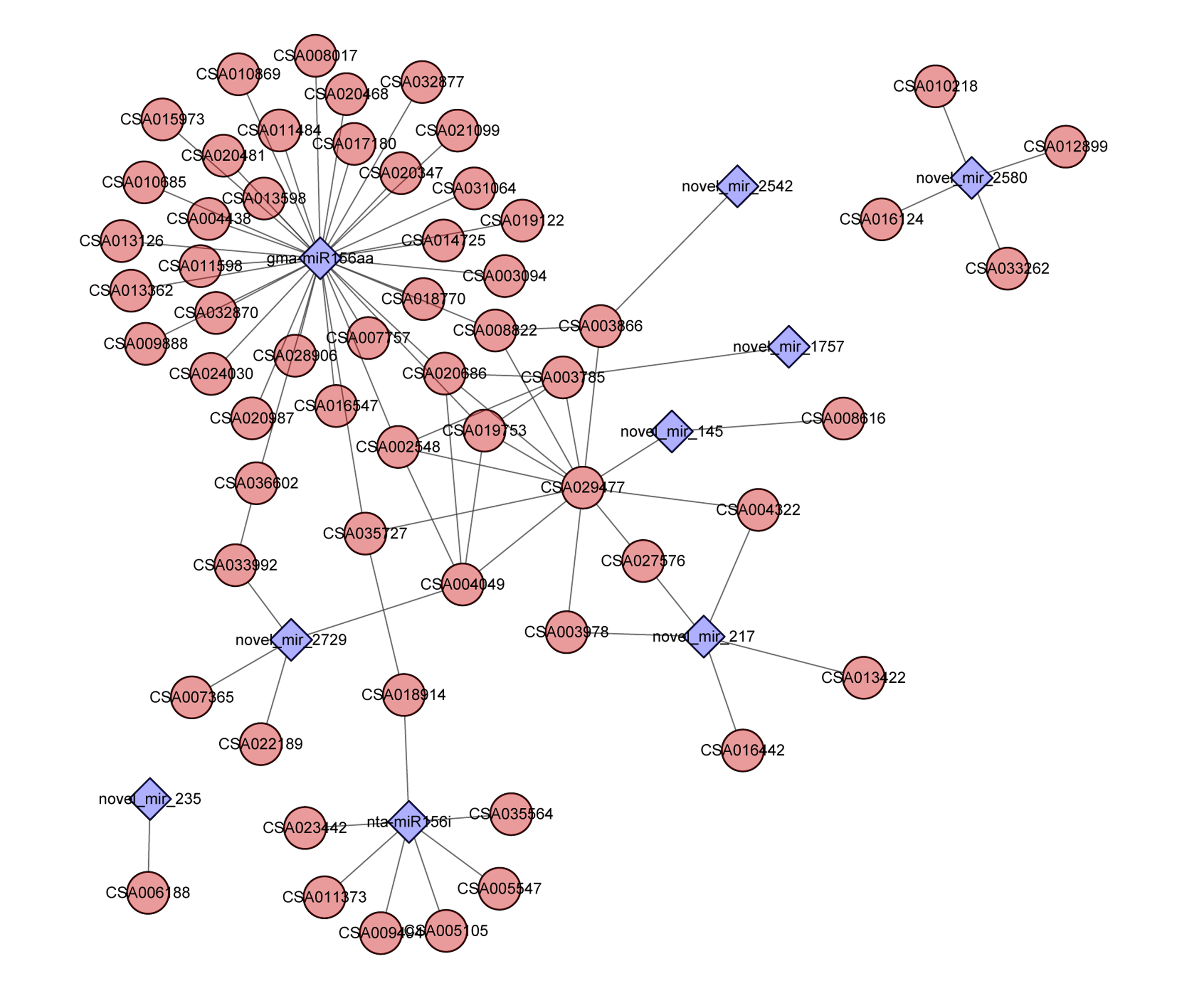

Supplement: Supplementary file 1 — Figure S1 [file 41438_2020_290_MOESM1_ESM.tif]

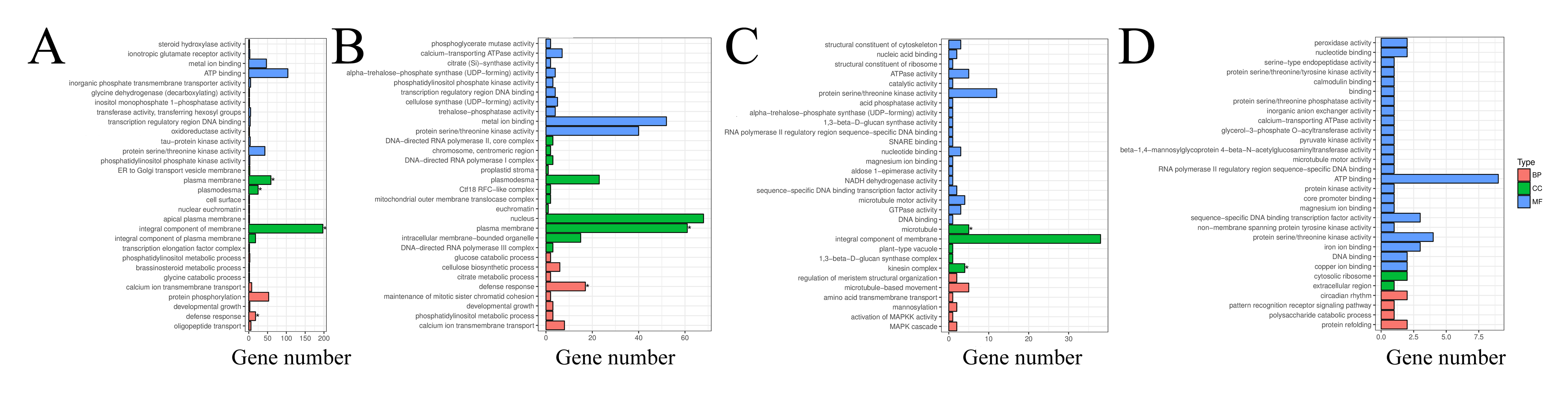

Supplement: Supplementary file 2 — Figure S2 [file 41438_2020_290_MOESM2_ESM.tif]
