# Supplementary material for: Integrative transcriptome, proteome, and microRNA analysis reveals the effects of nitrogen sufficiency and deficiency conditions on theanine metabolism in the tea plant (Camellia sinensis)
Source: Hortic Res. 2020 May 1;7:65. doi: 10.1038/s41438-020-0290-8 (PMC7192918; doi:10.1038/s41438-020-0290-8)
Supplement: Supplementary file 3 — Table S1–11 [file 41438_2020_290_MOESM3_ESM.doc]

**Table S1** The primers for RT-qPCR of DEGs involved in theanine metabolism.

| Gene name | Gene ID | Forward primer (5’-3’) | Reverse primer (5’-3’) |
| --- | --- | --- | --- |
| *NiR* | CSA002229 | AGAGGTTCGCAGGGTATG | AAACAAGTAGTGCTCCAACAG |
| *NRT* | CSA018684 | TAGTGTTCTTTTCGGGCTCA | AACCCTTCTGTTTCTCCTCATC |
| *NRT* | CSA029669 | CGAGGCATTTATGTCTATTG | GCTCAGATAGCTTGATACCG |
| *GOGAT* | CSA003654 | TGCTGGACAGTCATTTGC | ATTCACAACAATGGTCGC |
| *GDH* | CSA011735 | ATTCAAGGTTTTATGTGGGACG | AGCCCGAGCAACACGATT |
| *GAD* | CSA021427 | GTCCTTCGTGTTGTCGTG | CAGGCTTGTATTGCTTCC |
| *GLR* | CSA013368 | CATTAGGGACCAAGACGA | CCAACCTTTTGAGGGAAC |
| *GLR* | CSA026127 | TTACTAAAAGCGGATGGGG | AACTTGGTTGCCTTGTGGG |
| *ALT* | CSA034512 | TTGGAGACGAGTCCTACG | TTCTGCTGCCTCTATTGC |
| *ADC* | CSA021727 | GTATTGGGCAGCTTTTCC | AACACGCACGACACTTGG |
| *SAMDC* | CSA012900 | ACTGAGTTCTCCATTGCTATAC | TTCTCGTCATCCTCGTTC |
| *pAO* | CSA032875 | GAGTTTCCAAATCAAAATCCCC | CACCACCACATTGTCCTTCAC |
| *pAO* | CSA016220 | CGAGTCGGAAGCAAAAGC | ACCTGTCATAAGGGGTCAC |
| *PEPC* | CSA028217 | CGTAGCAGGAACAGAGGA | CCGAGTGTATCGCATCAA |
| *PK* | CSA012967 | CCAACTCAGCGAAAGCAA | TGTGAAGCCCTAGCAGACC |
| *PK* | CSA011300 | GCGGGCTGCTATTACAGT | TCTTGAGACGAGGGATGA |
| *AMT* | CSA023496 | ACATGCCATCGCAGGAAG | CCCAATTTGTCGAAACCCTA |
| *AQP* | CSA031752 | TTCACCTTGCCACCATCC | GGTCCAACCCAAAACATCC |
| *PDC* | CSA032830 | GCAAAGGCAACTGGGGAGA | GGAGGCGACTATTGGCAGA |
| *PDH-E1* | CSA024211 | TTCGAGCACGTTTTGCTTTAC | ACTTCGGGATCATACCCTTTG |
| *PDH-E2* | CSA005663 | GTGGCACTTTCAGCATTTCG | GGCTTCTCGATTCCATCAGTT |
| *PDH-E2* | CSA004322 | TCAGCACCCAGTTGTGAATAC | TGCCCAAATTGGAAAGAGTA |
| *PDP* | CSA033992 | GGTATTTGGACTTGAAGAAGATCG | TCACGGACAGGTTGGGACTC |
| *PDP* | CSA014790 | TGATGACTTTGCCGTTGAA | GTGGAAATGCCGCCTAAC |
| *PDP* | CSA003785 | TGCCTATCTTGTCTGCTACCC | AGCCTCTTCGCACTTCCTG |
| *PDK* | CSA022898 | GGTAGCAAGATGTGCCAGTG | CCATAAACCGCTCCTCGA |
| *NRT* | CSA018682 | TTTGTGAATTTTCCCCAGTG | TTCTCCTGCTCGCTCCACTC |
| *PEPC* | CSA019988 | ATTGCTGGGCACAAGGAT | TTTGGGGCTTGCTAGTAGAGT |
| *AQP* | CSA016542 | CTGTGATGGGTTGGGCTTAT | TCTTCTTTGGGTGGTTGGAC |
| *PDK* | CSA006579 | GGTAGCAAGATGTGCCAGTG | CCATAAACCGCTCCTCGA |
| *PK* | CSA032877 | AGCAGTCGGATGGAACTATGG | ACGGTTGCGTGAGAAGAGG |

**Table S2** The primers forRT-qPCR of DEGs-DEMs involved in theanine metabolism.

| miRNA ID | Forward primer | Gene ID | Forward primer (5’-3’) | Reverse primer (5’-3’) | Gene name |
| --- | --- | --- | --- | --- | --- |
| novel_mir_145 | CAAAGTGATCCATACCATTCA | CSA029477 | GCAGCGAGGAGATGATTGA | CACCTTCCACATTGGTTGAG | *GOGAT* |
| novel_mir_235 | ACAAACCCTACAAAATGAACGG | CSA006188 | AATCTGTCACTGATACCCACC | CAGCCATCGGAGTTTCTT | *GLR* |
| gma-miR156aa | ATTGGAGTGAAGGGAGCT | CSA020347 | TCTTGGGCCTGTTTCTCAT | ACGGGTCTTGCTTCTCGTC | *GLR* |
| nta-miR156i | TGACAGAAGATAGAGAGCAC | CSA005105 | AAGCATTGCGGCTAGGAC | ATTAGGCTCTGGGGTTTT | *GLR* |
| novel_mir_217 | CCAACTCCTTTCATTTCTCTG | CSA004322 | TCAGCACCCAGTTGTGAA | TTTCTACCAACTCCTTCCAT | *PDH-E2* |
| gma-miR156aa | ATTGGAGTGAAGGGAGCT | CSA032877 | CAGTCGGATGGAACTATGG | ACGGTTGCGTGAGAAGAGG | *PK* |
| novel_mir_2542 | GATCCGAACCGTTCATATTTT | CSA003866 | GGACAGGCTGCCCAAATAG | TCACTCCCAATCCTCCCTC | *NiR* |
| novel_mir_2580 | GAAGCTCAGATCTGTGTGTT | CSA012899 | TTCCAGCCAAGCGAGTTC | AACCTCCCTCCTTGCCGA | *SAMDC* |
| novel_mir_2729 | GCTCCCGGAGGCCGTCCGAGA | CSA033992 | GGTATTTGGACTTGAAGAAGATCG | TCACGGACAGGTTGGGACTC | *PDP* |
| novel_mir_1757 | TCTGCACATCTTTTCGTAAAAA | CSA003785 | TGCCTATCTTGTCTGCTACC | CGAATATCGCATCTCCCT | *PDP* |

**Table S3** The all and differentially expressed miRNA-mRNA pairs statistics.

|  | Number | DE miRNA-mRNA pairs in N^6-^R/N^6-^S | DE miRNA-mRNA pairs in N^6-^R/N^6+^R | DE miRNA-mRNA pairs in N^6-^S/N^6+^S | DE miRNA-mRNA pairs in N^6+^R/N^6+^S | All miRNA-mRNA pairs |
| --- | --- | --- | --- | --- | --- | --- |
| Known | miRNA | 1574 | 475 | 540 | 1686 | 5176 |
|  | Target mRNA | 2891 | 1100 | 830 | 2653 | 12329 |
|  | pairs | 20996 | 7408 | 6030 | 20653 | 52553 |
| novel | miRNA | 457 | 277 | 742 | 563 | 2402 |
|  | Target mRNA | 2233 | 1582 | 2708 | 2445 | 7223 |
|  | pairs | 3251 | 2077 | 4514 | 3816 | 15359 |

**Table S4** The DEGs and corresponding miRNAs involved in theanine metabolism.

| **Group** | **N^6-^R/N^6-^S** | | | **N^6+^R/N^6+^S** | | | **N^6-^R/N^6+^R** | | | **N^6-^S/N^6+^S** | | |
| --- | --- | --- | --- | --- | --- | --- | --- | --- | --- | --- | --- | --- |
| **Gene name** | **Gene ID** | **miRNA ID** | **Regulation** | **Gene ID** | **miRNA ID** | **Regulation** | **Gene ID** | **miRNA ID** | **Regulation** | **Gene ID** | **miRNA ID** | **Regulation** |
| ***NR*** | CSA020650 | - | Down |  |  |  | CSA020650 | - | Down |  |  |  |
| ***NiR*** | CSA003866 | novel_mir_2542 | Up | CSA003866 | novel_mir_2542 | Up | CSA020650 | - | Down | CSA003866 | novel_mir_2542 | Down |
|  | CSA020650 | - | Down |  |  |  |  |  |  |  |  |  |
| ***NRT*** | CSA011051 | - | Up | CSA011051 | - | Up | CSA034528 | - |  | CSA006476 | - | Up |
|  | CSA034528 | - | Up | CSA006476 | - | Up | CSA034045 | cre-miR906-3p |  | BGI_novel_G005965 | - | Up |
|  | CSA034045 | cre-miR906-3p | Up | CSA020711 | novel_mir_144 | Up | CSA029669 | - |  |  |  |  |
|  | CSA006476 | - | Up | CSA034528 | - | Up |  |  |  |  |  |  |
|  | CSA020711 | novel_mir_144 | Up | CSA034045 | cre-miR906-3p | Up |  |  |  |  |  |  |
|  | CSA029669 | - | Down | BGI_novel_G005965 | - | Up |  |  |  |  |  |  |
|  | CSA024363 | - | Up | CSA029669 | - | Down |  |  |  |  |  |  |
|  | CSA011542 | novel_mir_1961 | Down | CSA011542 | novel_mir_1961 | Down |  |  |  |  |  |  |
| ***GS*** | BGI_novel_G000705 | - | Up | BGI_novel_G000705 | - | Up |  |  |  | CSA006044 | - | Up |
|  | CSA030118 | ppe-miR7122b-3p | Up | CSA030118 | ppt-miR1044-3p | Up |  |  |  |  |  |  |
|  | CSA001114 | gma-miR171i-5p | Down | CSA001114 | gma-miR171i-5p | Down |  |  |  |  |  |  |
| ***GOGAT*** | CSA029477 | novel_mir_645 | Up | CSA029477 | novel_mir_645 | Up |  |  |  |  |  |  |
|  | BGI_novel_G009129 | - | Down |  |  |  |  |  |  |  |  |  |
|  | CSA003654 | gma-miR1522 | Down |  |  |  |  |  |  |  |  |  |
| ***GDH*** | CSA011735 | - | Up | CSA011735 | - | Up | CSA011735 | - | Up |  |  |  |
|  | CSA023959 | - | Up | CSA023959 | - | Up |  |  |  |  |  |  |
| ***GAD*** | CSA004788 | novel_mir_1052 | Up | CSA025312 | - | Up |  |  |  |  |  |  |
|  | CSA025312 | - | Up | BGI_novel_G009175 | - | Up |  |  |  |  |  |  |
|  |  |  |  | CSA025313 | - | Up |  |  |  |  |  |  |
| ***AS*** | CSA035384 | novel_mir_805 | Up | CSA035384 | novel_mir_805 | Up | CSA035384 | novel_mir_805 | Up | CSA035384 | novel_mir_805 | Up |
| ***GLR*** | BGI_novel_G006089 | - | Up | BGI_novel_G006089 | - | Up | BGI_novel_G006210 | - | Up |  |  |  |
|  | BGI_novel_G000639 | - | Up | CSA026178 | ptc-miR6431 | Up | CSA002720 | aly-miR4248c | Up |  |  |  |
|  | BGI_novel_G006210 | - | Up | BGI_novel_G006088 | - | Up | BGI_novel_G000639 | - | Up |  |  |  |
|  | CSA026178 | ptc-miR6431 | Up | BGI_novel_G009926 | - | Up | CSA013368 | osa-miR2105 | Up |  |  |  |
|  | BGI_novel_G006088 | - | Up | CSA006188 | novel_mir_1433 | Up | CSA006188 | novel_mir_1433 | Up |  |  |  |
|  | CSA002720 | aly-miR4248b | Up | CSA013368 | gra-miR8668 | Up | CSA020347 | zma-miR399d-3p | Down |  |  |  |
|  | BGI_novel_G009926 | - | Up | CSA018952 | - | Up |  |  |  |  |  |  |
|  | CSA006188 | novel_mir_235 | Up | CSA026127 | tae-miR9670-3p | Down |  |  |  |  |  |  |
|  | CSA013368 | ath-miR5656 | Up | CSA026128 | stu-miR172c-3p | Down |  |  |  |  |  |  |
|  | CSA018952 | - | Up | CSA014148 | novel_mir_1588 | Down |  |  |  |  |  |  |
|  | CSA026127 | novel_mir_1896 | Down | CSA005105 | aly-miR157d-5p | Down |  |  |  |  |  |  |
|  | CSA020347 | gma-miR399a | Down |  |  |  |  |  |  |  |  |  |
|  | CSA014148 | novel_mir_1588 | Down |  |  |  |  |  |  |  |  |  |
|  | CSA026128 | stu-miR172c-3p | Down |  |  |  |  |  |  |  |  |  |
|  | CSA005105 | mdm-miR156x | Down |  |  |  |  |  |  |  |  |  |
| ***ALT*** | CSA034512 | mtr-miR5748 | Up | CSA034512 | mtr-miR5748 | Up |  |  |  |  |  |  |
| ***ADC*** | CSA021727 | novel_mir_1416 | Up | CSA021727 | novel_mir_1416 | Up |  |  |  |  |  |  |
|  | CSA010790 | - | Up | CSA010790 | - | Up |  |  |  |  |  |  |
| ***SAMDC*** | CSA012900 | novel_mir_589 | Up | CSA012900 | novel_mir_589 | Up | CSA012900 | novel_mir_589 | Up |  |  |  |
|  | CSA012899 | novel_mir_2580 | Up | CSA012899 | novel_mir_2580 | Up |  |  |  |  |  |  |
|  | CSA005827 | - | Up | CSA005827 | - | Up |  |  |  |  |  |  |
|  | CSA035590 | - | Down |  |  |  |  |  |  |  |  |  |
| ***PAO*** | CSA016220 | novel_mir_1562 | Down | CSA015998 | - | Up | CSA009339 | gma-miR156z | Up |  |  |  |
|  | CSA015927 | - | Down | CSA009339 | gma-miR156z | Down | CSA016220 | novel_mir_1562 | Down |  |  |  |
|  | CSA009339 | gma-miR156aa | Down | CSA015927 | - | Down |  |  |  |  |  |  |
|  | CSA034879 | novel_mir_2150 | Down |  |  |  |  |  |  |  |  |  |
| ***PEPC*** | CSA032884 | - | Up | CSA011564 | mtr-miR5230 | Up | CSA032884 | - | Up | CSA005647 | nd | Up |
|  | CSA028217 | - | Up | CSA019990 | rgl-miR7797a | Down | CSA019988 | - | Up | CSA011564 | mtr-miR5230 | Up |
|  | CSA011564 | mtr-miR5230 | Up | CSA005647 | - | Down |  |  |  |  |  |  |
|  | CSA019990 | rgl-miR7797a | Down | CSA005645 | - | Down |  |  |  |  |  |  |
|  | CSA005647 | - | Down |  |  |  |  |  |  |  |  |  |
|  | CSA005645 | - | Down |  |  |  |  |  |  |  |  |  |
| ***PK*** | CSA012967 | - | Up | CSA012967 | - | Up |  |  |  | CSA032877 | mtr-miR5751 | Up |
|  | CSA025782 | - | Down | CSA024935 | ppt-miR1054 | Up |  |  |  |  |  |  |
|  | CSA011300 | - | Down | CSA034624 | hvu-miR6189 | Up |  |  |  |  |  |  |
|  |  |  |  | CSA018123 | - | Up |  |  |  |  |  |  |
|  |  |  |  | CSA019642 | sly-miR9479-3p | Up |  |  |  |  |  |  |
|  |  |  |  | BGI_novel_G006775 | - | Down |  |  |  |  |  |  |
|  |  |  |  | CSA011300 | - | Down |  |  |  |  |  |  |
|  |  |  |  | CSA025782 | - | Down |  |  |  |  |  |  |
| ***AMT*** | CSA025550 | - | Up | CSA025550 | - | Up | CSA031722 | - | Up |  |  |  |
|  | CSA022902 | - | Up | CSA022902 | - | Up | CSA025550 | - | Up |  |  |  |
|  | CSA011804 | - | Up | CSA028289 | - | Up |  |  |  |  |  |  |
|  | CSA028289 | - | Up | CSA036049 | novel_mir_2264 | Up |  |  |  |  |  |  |
|  | CSA031722 | - | Up | CSA011804 | - | Up |  |  |  |  |  |  |
|  | CSA023343 | - | Up | CSA023775 | gra-miR172a | Up |  |  |  |  |  |  |
|  | CSA018541 | novel_mir_748 | Down | CSA018541 | novel_mir_748 | Down |  |  |  |  |  |  |
| ***AQP*** | CSA014714 | - | Up | CSA000157 | - | Up | CSA016542 | novel_mir_32 | Up | CSA000157 | nd | Up |
|  | CSA004835 | - | Up | CSA014714 | - | Up | CSA030184 | ppt-miR893 | Up | CSA015173 | nd | Down |
|  | CSA000157 | - | Up | BGI_novel_G003868 | - | Up | CSA014714 | - | Up | CSA036134 | nd | Down |
|  | CSA015173 | - | Up | CSA025076 | - | Up | CSA018859 | - | Up |  |  |  |
|  | CSA036134 | - | Up | CSA015173 | - | Up | BGI_novel_G003868 | - | Up |  |  |  |
|  | BGI_novel_G003868 | - | Up | CSA036134 | - | Up | CSA015173 | - | Up |  |  |  |
|  | CSA025076 | - | Up | CSA013669 | - | Up | CSA028896 | - | Up |  |  |  |
|  | CSA013669 | - | Up | CSA023001 | novel_mir_32 | Up | CSA017078 | - | Up |  |  |  |
|  | CSA017078 | - | Up | CSA017078 | - | Up | CSA025076 | - | Up |  |  |  |
|  | BGI_novel_G004130 | - | Up | CSA004834 | hbr-miR6482 | Up | CSA036134 | - | Up |  |  |  |
|  | CSA023001 | novel_mir_32 | Up | CSA011972 | hbr-miR6482 | Up | CSA011041 | - | Down |  |  |  |
|  | CSA024539 | - | Up | BGI_novel_G004130 | - | Up |  |  |  |  |  |  |
|  | CSA020058 | - | Up | CSA030184 | ppt-miR893 | Down |  |  |  |  |  |  |
|  | CSA011972 | hbr-miR6482 | Up | CSA030470 | - | Down |  |  |  |  |  |  |
|  | CSA004834 | hbr-miR6482 | Up | CSA011041 | - | Down |  |  |  |  |  |  |
|  | CSA031752 | - | Up |  |  |  |  |  |  |  |  |  |
|  | CSA030184 | ppt-miR893 | Down |  |  |  |  |  |  |  |  |  |
|  | CSA011041 | - | Down |  |  |  |  |  |  |  |  |  |
|  | CSA027609 | novel_mir_2557 | Down |  |  |  |  |  |  |  |  |  |
|  | CSA030470 | - | Down |  |  |  |  |  |  |  |  |  |
|  | BGI_novel_G007770 | - | Down |  |  |  |  |  |  |  |  |  |
| ***NtrB*** | CSA009523 | - | Down |  |  |  |  |  |  |  |  |  |
| ***PDC*** | CSA016176 | mtr-miR5206b | Up | CSA016176 | mtr-miR5206b | Up |  |  |  |  |  |  |
|  |  |  |  | CSA002466 | - | Up |  |  |  |  |  |  |
| ***PDH-E1*** | CSA007666 | gra-miR8718 | Up | CSA007666 | gra-miR8718 | Up |  |  |  |  |  |  |
|  | CSA032487 | ath-miR773b-3p | Up | BGI_novel_G002249 | - | Up |  |  |  |  |  |  |
|  | BGI_novel_G002249 | - | Up | CSA004843 | bdi-miR169e-3p | Down |  |  |  |  |  |  |
|  | CSA004843 | bdi-miR169e-3p | Down |  |  |  |  |  |  |  |  |  |
| ***PDH-E2*** | BGI_novel_G006357 | - | Up |  |  |  |  |  |  |  |  |  |
|  | CSA005663 | gra-miR7494c | Up | CSA005663 | gra-miR7494c | Up |  |  |  |  |  |  |
|  | CSA033762 | - | Up | CSA033762 | - | Up |  |  |  |  |  |  |
|  | CSA004322 | novel_mir_217 | Down |  |  |  |  |  |  |  |  |  |
| ***PDP*** | CSA013926 | - | Up | CSA000307 | - | Up |  |  |  | CSA024803 | nd | Up |
|  | CSA033992 | novel_mir_2729 | Up | BGI_novel_G004020 | - | Up |  |  |  | CSA003785 | novel_mir_1757 | Up |
|  | BGI_novel_G004020 | - | Up | CSA024803 | - | Up |  |  |  | CSA000307 | nd | Up |
|  | CSA004049 | novel_mir_2729 | Up | CSA014790 | - | Up |  |  |  |  |  |  |
|  | CSA001205 | - | Up | CSA004049 | novel_mir_2729 | Up |  |  |  |  |  |  |
|  | CSA014790 | - | Up | CSA033992 | novel_mir_2729 | Up |  |  |  |  |  |  |
|  | CSA000307 | - | Up | CSA027012 | - | Up |  |  |  |  |  |  |
|  | CSA024803 | - | Up | CSA014860 | - | Up |  |  |  |  |  |  |
|  | CSA026779 | - | Up | BGI_novel_G006343 | - | Down |  |  |  |  |  |  |
|  | CSA003785 | novel_mir_1757 | Down |  |  |  |  |  |  |  |  |  |
| ***PDK*** | CSA006579 | - | Up | CSA006579 |  | Up | CSA022898 | gma-miR9755 | Up |  |  |  |
|  | CSA022898 | gma-miR9755 | Up |  |  |  | CSA006579 | - | Up |  |  |  |

**Table S5** The number statistics of DEGs involved in theanine metabolism pathway.

| Gene name | N^6-^R/N^6+^R | | N^6-^S/N^6+^S | | N^6+^R/N^6+^S | | N^6-^R/N^6-^S | |
| --- | --- | --- | --- | --- | --- | --- | --- | --- |
|  | up | down | up | down | up | down | up | down |
| *NR* | - | 1 | - | - | - | - | - | 1 |
| *NiR* | - | 1 | - | 1 | 1 | - | 1 | 1 |
| *NRT* | 3 | - | 2 | - | 6 | 2 | 6 | 2 |
| *GS* | - | - | 1 | - | 2 | 1 | 2 | 1 |
| *GOGAT* | - | - | - | - | 1 | - | 1 | 2 |
| *GDH* | 1 | - | - | - | 2 | - | 2 | - |
| *GAD* | - | - | - | - | 3 | - | 2 | - |
| *AS* | 1 | - | 1 | - | - | - | 1 | - |
| *GMPS* | - | - | - | - | 1 |  | - | - |
| *GLR* | 5 | 1 | - | - | 7 | 4 | 10 | 5 |
| *ALT* | - | - | - | - | 1 | - | 1 | - |
| *ADC* | - | - | - | - | 2 | - | 2 | - |
| *SAMDC* | 1 | - | - | - | 3 | - | 3 | 1 |
| *pAO* | 1 | 1 | - | - | 1 | 2 | - | 4 |
| *PEPC* | - | - | 2 | - | 1 | 3 | 3 | 3 |
| *PK* | - | - | 1 | - | 5 | 3 | 1 | 2 |
| *AMT* | 2 | - | - | - | 6 | 1 | 6 | 1 |
| *AQP* | 10 | 1 | 1 | 2 | 12 | 3 | 16 | 5 |
| *GlnB* | - | - | - | - | - | - | - | 1 |
| *PDC* | - | - | - | - | 2 | - | 1 | - |
| *PDH-E1* | - | - | - | - | 2 | 1 | 3 | 1 |
| *PDH-E2* | - | - | - | - | 2 | - | 3 | 1 |
| *PDP* | - | - | 3 | - | 8 | 1 | 9 | 1 |
| *PDK* | 2 | - | - | - | 1 | - | 2 | - |

-: not found

**Table S6** The regulation validation of DEGs involved in theanine metabolism.

| Group | Gene name | Gene ID | Illumina mRNA-seq  (log2 FC) | Regulation | RT-PCR  (log2 FC) |
| --- | --- | --- | --- | --- | --- |
| N^6-^R/N^6-^S | *NiR* | CSA002229 | -4.19 | down | -2.45 |
|  | *NRT* | CSA018684 | 11.80 | up | 17.28 |
|  |  | CSA029669 | -5.15 | down | -1.77 |
|  | *GOGAT* | CSA003654 | -1.39 | down | -3.92 |
|  | *GDH* | CSA011735 | 9.24 | up | 4.38 |
|  | *GAD* | CSA021427 | -2.53 | down | -4.1 |
|  | *GLR* | CSA013368 | 6.25 | up | 13.41 |
|  |  | CSA026127 | -4.31 | down | -3.03 |
|  | *ALT* | CSA034512 | 1.79 | up | 1.79 |
|  | *ADC* | CSA021727 | 2.06 | up | 1.91 |
|  | *SAMDC* | CSA012900 | 13.61 | up | 3.56 |
|  | *PAO* | CSA032875 | 3.45 | up | 5.85 |
|  |  | CSA016220 | -4.02 | down | -2.08 |
|  | *PEPC* | CSA028217 | 3.76 | up | 4.04 |
|  | *PK* | CSA012967 | 1.68 | up | 1.43 |
|  |  | CSA011300 | -4.18 | down | -9.05 |
|  | *AMT* | CSA023496 | 7.76 | up | 8.37 |
|  | *AQP* | CSA031752 | 1.09 | up | 1.56 |
|  | *PDC* | CSA032830 | -6.69 | down | -6.03 |
|  | *PDH-E1* | CSA024211 | -1.28 | down | -1.58 |
|  | *PDH-E2* | CSA005663 | 1.31 | up | 3.03 |
|  |  | CSA004322 | -1.26 | down | -1.85 |
|  | *PDP* | CSA033992 | 2.89 | up | 2.34 |
|  |  | CSA014790 | 1.51 | up | 2.37 |
|  |  | CSA003785 | -1.15 | down | -1.01 |
|  | *PDK* | CSA022898 | 1.38 | up | 0.91 |
| N^6+^R/N^6+^S | *NRT* | CSA018684 | 8.28 | up | 8.96 |
|  |  | CSA029669 | -6.25 | down | -2.16 |
|  | *GOGAT* | CSA003654 | -2.13 | down | -2.99 |
|  | *GDH* | CSA011735 | 7.99 | up | 4.51 |
|  | *GAD* | CSA021427 | -1.51 | down | -1.33 |
|  | *GLR* | CSA013368 | 4.20 | up | 2.71 |
|  |  | CSA026127 | -5.09 | down | -7.13 |
|  | *ALT* | CSA034512 | 2.85 | up | 5.51 |
|  | *ADC* | CSA021727 | 2.18 | up | 2.67 |
|  | *SAMDC* | CSA012900 | 10.24 | up | 5.73 |
|  | *PAO* | CSA032875 | 2.55 | up | 2.97 |
|  | *PK* | CSA012967 | 2.14 | up | 2.17 |
|  |  | CSA011300 | -3.35 | down | -5.49 |
|  | *AMT* | CSA023496 | 7.15 | up | 4.55 |
|  | *PDH-E1* | CSA024211 | -1.43 | down | -2.50 |
|  | *PDH-E2* | CSA005663 | 1.19 | up | 2.14 |
|  | *PDP* | CSA033992 | 1.60 | up | 1.67 |
|  |  | CSA014790 | 2.01 | up | 2.22 |
| N^6-^R/N^6+^R | *NRT* | CSA018684 | 1.94 | up | 2.65 |
|  |  | CSA018682 | -1.19 | down | -2.51 |
|  | *GDH* | CSA011735 | 1.22 | up | 2.52 |
|  | *GAD* | CSA021427 | -1.02 | down | -2.35 |
|  | *GLR* | CSA013368 | 3.28 | up | 2.70 |
|  |  | CSA020347 | -1.12 | down | -2.31 |
|  | *SAMDC* | CSA012900 | 3.36 | up | 2.65 |
|  | *PAO* | CSA016220 | -2.79 | down | -2.45 |
|  | *PEPC* | CSA019988 | 1.50 | up | 1.47 |
|  | *AQP* | CSA016542 | 8.91 | up | 6.56 |
|  | *PDK* | CSA006579 | 1.08 | up | 2.62 |
| N^6-^S/N^6+^S | *PK* | CSA032877 | 1.14 | up | 2.50 |
|  | *PDC* | CSA032830 | 2.04 | up | 2.67 |
|  | *PDP* | CSA003785 | 1.02 | up | 2.06 |

**Table S7** The number statistics of DEPs-DEGs correlation pairs.

| Group | P-up_T-up | P-down_T-down | P-up_T-down | P-down_T-up | all |
| --- | --- | --- | --- | --- | --- |
| N^6-^R/N^6+^R | 2 | 3 | 1 | 2 | 8 |
| N^6-^S/N^6+^S | 1 | - | - | - | 1 |
| N^6+^R/N^6+^S | 68 | 48 | 8 | 14 | 138 |
| N^6-^R/N^6-^S | 75 | 59 | 10 | 13 | 157 |

P-up_T-up: ProteinUp-mRNAUp; P-down_T-down: ProteinDown-mRNADown; P-up_T-down: ProteinUp-mRNADown; P-down_T-up: ProteinDown-mRNAUp; -: none

**Table S8** The negative interactions of DEGs-DEMs involved in theanine metabolism.

| Group | miRNA ID | mRNA ID | Correlation coefficient | miRNA | | | mRNA | | | Gene name |
| --- | --- | --- | --- | --- | --- | --- | --- | --- | --- | --- |
|  |  |  |  | Illumina miRNA-seq  (log2 FC) | Regulation | RT-PCR  (log2 FC) | Illumina mRNA-seq  (log2 FC) | Regulation | RT-PCR  (log2 FC) |  |
| N^6-^R/N^6-^S | novel_mir_145 | CSA029477 | -0.44 | 12.17 | up | 4.56 | 2.42 | up | 2.79 | *GOGAT* |
|  | novel_mir_235 | CSA006188 | -0.34 | 6.88 | up | 4.96 | 6.81 | up | 2.48 | *GLR* |
|  | gma-miR156aa | CSA020347 | -0.15 | 7.95 | up | 3.86 | -2.35 | down | -4.02 |  |
|  | nta-miR156i | CSA005105 | -0.09 | 2.82 | up | 1.79 | -1.05 | down | -1.89 |  |
|  | novel_mir_217 | CSA004322 | -0.13 | 6.59 | up | 4.43 | -1.26 | down | -1.21 | *PDH-E2* |
| N^6+^R/N^6+^S | novel_mir_145 | CSA029477 | -0.44 | 12.36 | up | 10.21 | 3.25 | up | 1.96 | *GOGAT* |
|  | novel_mir_235 | CSA006188 | -0.34 | -6.61 | down | -7.00 | 5.04 | up | 9.36 | *GLR* |
|  | nta-miR156i | CSA005105 | -0.09 | 1.29 | up | 2.51 | -1.12 | down | -3.45 |  |
|  | novel_mir_2542 | CSA003866 | -0.22 | 5.05 | up | 3.33 | 1.15 | up | 2.27 | *NiR* |
|  | novel_mir_2580 | CSA012899 | -0.56 | 6.43 | up | 7.82 | 3.03 | up | 4.16 | *SAMDC* |
|  | novel_mir_2729 | CSA033992 | -0.47 | 7.05 | up | 6.30 | 1.60 | up | 0.93 | *PDP* |
| N^6-^R/N^6+^R | novel_mir_235 | CSA006188 | -0.34 | 5.58 | up | 8.08 | 2.83 | up | 3.15 | *GLR* |
| N^6-^S/N^6+^S | gma-miR156aa | CSA032877 | -0.86 | 2.08 | up | 1.93 | 1.14 | up | 1.95 | *PK* |
|  | novel_mir_1757 | CSA003785 | -0.31 | 4.67 | up | 2.44 | 1.02 | up | 1.79 | *PDP* |

FC: Fold Change

**Table S9** The contents of seventeen free amino acids in tea plant (mg/100g).

| Name | N^0^S | N^0^O | N^0^R | N^3+^S | N^3+^O | N^3+^R | N^3-^S | N^3-^O | N^3-^R | N^6+^S | N^6+^O | N^6+^R | N^6-^S | N^6-^O | N^6-^R | N^9+^S | N^9+^O | N^9+^R | N^9-^S | N^9-^O | N^9-^R |
| --- | --- | --- | --- | --- | --- | --- | --- | --- | --- | --- | --- | --- | --- | --- | --- | --- | --- | --- | --- | --- | --- |
| Thea | 62.83 | 1.14 | 342.25 | 53.13 | 2.83 | 286.20 | 61.00 | 1.33 | 362.25 | 233.60 | 10.17 | 486.10 | 113.75 | 2.17 | 369.10 | 203.90 | 6.33 | 455.30 | 69.50 | 1.17 | 457.00 |
| Glu | 238.06 | 156.03 | 167.81 | 259.24 | 178.04 | 153.24 | 209.65 | 166.43 | 172.33 | 386.14 | 201.73 | 163.14 | 326.69 | 191.16 | 163.64 | 445.48 | 170.45 | 152.50 | 294.60 | 150.42 | 139.10 |
| Ala | 16.97 | 4.78 | 19.14 | 16.43 | 8.70 | 24.45 | 13.49 | 6.87 | 26.32 | 23.67 | 6.77 | 19.75 | 15.74 | 4.60 | 13.52 | 24.75 | 6.48 | 18.24 | 17.10 | 7.05 | 27.11 |
| Arg | 17.39 | 9.32 | 142.39 | 14.07 | 4.32 | 152.96 | 12.81 | 9.40 | 158.33 | 53.35 | 9.70 | 211.06 | 13.23 | 9.14 | 183.58 | 55.69 | 9.39 | 224.70 | 19.85 | 20.47 | 167.98 |
| Val | 7.86 | 4.48 | 13.03 | 8.54 | 4.71 | 15.13 | 6.70 | 6.62 | 15.92 | 5.83 | 4.34 | 12.27 | 10.00 | 5.29 | 14.32 | 6.96 | 5.07 | 10.43 | 6.27 | 4.96 | 12.2 |
| Leu | 5.61 | 2.08 | 10.89 | 6.95 | 1.84 | 14.92 | 6.58 | 7.67 | 14.38 | 6.06 | 2.29 | 14.17 | 8.14 | 2.72 | 15.01 | 9.90 | 2.93 | 9.79 | 4.62 | 2.70 | 15.02 |
| Asp | 101.53 | 42.17 | 156.08 | 136.10 | 59.00 | 139.58 | 109.80 | 65.45 | 162.10 | 175.21 | 93.91 | 146.89 | 169.47 | 92.07 | 150.20 | 198.47 | 107.55 | 142.66 | 323.29 | 85.76 | 128.05 |
| Thr | - | 16.69 | 34.41 | - | 16.09 | 79.44 | - | 21.67 | 81.26 | - | 17.03 | 83.72 | - | 19.92 | 93.46 | - | 18.52 | 75.45 | - | 21.80 | 67.32 |
| Lys | 9.56 | 8.31 | 12.90 | 11.03 | 2.69 | 16.84 | 10.17 | 9.71 | 16.58 | 12.48 | 8.10 | 18.76 | 12.57 | 2.77 | 17.89 | 15.44 | 9.36 | 16.47 | 10.90 | 9.27 | 16.09 |
| Met | 0.56 | - | 0.96 | 0.44 | - | 1.03 | 0.41 | - | 1.14 | 0.73 | - | 1.13 | 0.52 | - | 1.42 | 1.20 | - | 0.81 | 0.57 | - | 5.35 |
| Ile | 5.41 | 1.59 | 4.31 | 7.10 | 1.45 | 6.20 | 4.46 | 3.60 | 5.84 | 3.03 | 1.56 | 4.60 | 6.66 | 2.26 | 4.75 | 4.39 | 1.83 | 2.70 | 5.07 | 2.24 | 5.53 |
| Ser | 57.24 | 22.22 | 18.54 | 48.19 | 26.02 | 21.31 | 54.97 | 24.79 | 20.66 | 44.88 | 29.31 | 19.36 | 44.86 | 25.77 | 18.88 | 37.79 | 22.39 | 18.82 | 36.35 | 20.49 | 17.31 |
| Gly | 2.83 | 2.62 | 3.63 | 1.96 | 1.15 | 3.55 | 1.85 | 1.78 | 3.31 | 2.21 | 1.22 | 2.64 | 2.25 | 1.53 | 2.23 | 2.60 | 1.30 | 2.81 | 2.01 | 1.30 | 2.79 |
| Cys | 11.72 | 12.37 | 10.79 | 11.86 | 11.74 | 10.27 | 11.63 | 12.69 | 10.80 | 12.06 | 12.08 | 10.92 | 12.45 | 12.37 | 10.65 | 12.68 | 11.94 | 10.54 | 12.16 | 11.72 | 9.64 |
| His | 7.50 | 4.03 | 13.92 | 6.89 | 1.85 | 17.81 | 6.96 | 5.15 | 17.59 | 3.33 | 3.84 | 22.90 | 8.93 | 2.52 | 19.42 | 4.89 | 3.93 | 19.61 | 6.42 | 4.75 | 15.52 |
| Tyr | 8.58 | - | 7.87 | 10.51 | - | 8.88 | 11.67 | - | 9.15 | 9.86 | - | 9.18 | 10.11 | - | 9.41 | 9.54 | - | 8.02 | 8.25 | - | 9.76 |
| Phe | 26.46 | 20.72 | 24.90 | 31.80 | 17.48 | 33.83 | 25.42 | 25.63 | 32.39 | 30.88 | 20.82 | 31.05 | 31.32 | 18.94 | 30.25 | 35.56 | 24.37 | 29.45 | 32.40 | 21.74 | 30.58 |
| Total | 580.11 | 308.55 | 983.82 | 624.15 | 337.91 | 985.64 | 547.57 | 368.79 | 1110.35 | 1003.32 | 422.87 | 1257.64 | 786.69 | 393.23 | 1117.73 | 1069.24 | 401.84 | 1198.30 | 849.36 | 365.84 | 1126.35 |

**Table S10** The ID and FPKM values of genes involved in theanine metabolism.

| Gene name | Gene ID | Log_2_FPKM | | | |
| --- | --- | --- | --- | --- | --- |
|  |  | N^6-^S | N^6+^S | N^6-^R | N^6+^R |
| *SAMDCa* | CSA012899 | 5.9338087 | 5.9724629 | 9.4030333 | 9.0011267 |
| *AQP26* | CSA013669 | 6.5084287 | 6.0369428 | 9.3290339 | 8.8860928 |
| *GSb* | CSA014052 | 7.7667277 | 7.4346282 | 8.21757 | 7.8224752 |
| *ADCa* | CSA010790 | 7.054957 | 6.4034382 | 8.2256413 | 7.8667844 |
| *AQP15a* | CSA020058 | 6.2532329 | 6.5038257 | 7.7899248 | 7.3146059 |
| *PKa* | CSA024935 | 6.4202129 | 6.0578834 | 7.3620315 | 7.4061625 |
| *AQP19a* | CSA004834 | 6.3434078 | 6.4399555 | 7.5046204 | 7.8442978 |
| *AQP13a* | CSA025076 | 7.1946584 | 6.6722839 | 11.907991 | 10.827128 |
| *AQP7* | CSA011972 | 8.6209529 | 8.4893663 | 10.13277 | 9.7119422 |
| *AQP9a* | CSA031752 | 8.3621192 | 8.3075196 | 9.4524884 | 8.8635978 |
| *GSa* | CSA010785 | 8.7309795 | 8.9415172 | 8.2978334 | 8.7361651 |
| *pAOa* | CSA010476 | 9.3725604 | 9.5415032 | 9.4277738 | 8.8976337 |
| *AQP3* | CSA005896 | 9.5943619 | 9.2720932 | 9.2797731 | 8.758956 |
| *GLR3.3a* | CSA018952 | 2.3729521 | 2.3448285 | 3.797013 | 3.4854268 |
| *GADc* | CSA025312 | 1.9818527 | 2.0250288 | 3.7918141 | 4.1464923 |
| *PDP63c* | CSA004049 | 2.3219281 | 1.9818527 | 4.44228 | 3.9097731 |
| *GLR3.6* | CSA014148 | 4.3448285 | 3.9354597 | 2.6064422 | 2.7527486 |
| *AQP11c* | BGI_novel_G007770 | 4.0045014 | 3.4436067 | 2.3219281 | 3.1634987 |
| *PKchlG2* | BGI_novel_G002557 | 3.8359241 | 3.6064422 | 3.0071955 | 3.6724253 |
| *PDH-E2-5a* | CSA034852 | 3.8698714 | 3.502076 | 3.2898345 | 3.2854022 |
| *AMT3.1a* | BGI_novel_G009981 | 3.8589756 | 3.8914192 | 3.7676548 | 3.2311252 |
| *AMT3.1b* | CSA021032 | 4.0721058 | 4.0027025 | 3.2326608 | 3.0686708 |
| *PDP23c* | CSA025115 | 3.4659745 | 2.8953026 | 4.1009776 | 3.3840498 |
| *PDP25* | CSA002770 | 3.1276333 | 3.0373822 | 3.6610655 | 3.0391384 |
| *PDP42b* | BGI_novel_G004019 | 2.9183862 | 2.4620523 | 3.0531113 | 2.2016339 |
| *PKchlA* | CSA032877 | 3.4302853 | 2.2898345 | 3.0942361 | 3.165108 |
| *PDP42c* | CSA003785 | 3.892391 | 2.8718436 | 2.7463128 | 2.8479969 |
| *mtPDKb* | CSA022898 | 3.6461627 | 3.4763817 | 5.0254718 | 3.8991756 |
| *PKb* | CSA018123 | 3.351911 | 2.7990873 | 3.9754468 | 3.9504684 |
| *PDH-E2-1* | CSA005663 | 2.9708537 | 2.704872 | 4.2816983 | 3.892391 |
| *GDH1* | CSA009695 | 3.4607426 | 3.448901 | 4.2971914 | 4.129283 |
| *GMPSa* | CSA001747 | 3.5397792 | 3.2357271 | 3.7322692 | 3.8659188 |
| *PDP60d* | CSA010965 | 4.1399606 | 4.2794713 | 4.5185351 | 4.1505597 |
| *PDC1b* | CSA014147 | 4.6501901 | 3.7114949 | 4.3298412 | 4.0054 |
| *PDH-E1β3* | CSA024616 | 4.780835 | 4.0470148 | 4.4796186 | 4.0780974 |
| *GSd* | CSA006044 | 4.8948178 | 3.2341947 | 3.9836777 | 3.8288346 |
| *NADH-GOGATc* | BGI_novel_G009129 | 4.5891642 | 3.8489984 | 3.275007 | 3.8619554 |
| *NtrB* | CSA009523 | 4.741467 | 3.9927684 | 3.6123525 | 3.9126499 |
| *ASSchl* | CSA035466 | 4.22033 | 3.9364024 | 3.6252705 | 4.1546156 |
| *PDC2a* | CSA011615 | 4.4508813 | 4.3377111 | 3.5789387 | 4.0933912 |
| *PDC1a* | CSA016176 | 2.7718856 | 2.4059924 | 5.7564896 | 5.8906899 |
| *NRT2.5a* | CSA034045 | 1.6461627 | 1.7655347 | 5.6255627 | 4.3327079 |
| *AQP13b* | BGI_novel_G003868 | 1.9523336 | 0.8559897 | 6.7951955 | 5.3341392 |
| *PDP23a* | CSA000307 | 3.9049657 | 1.8359241 | 5.3402062 | 5.16551 |
| *ADCb* | CSA021727 | 3.0408924 | 2.7441611 | 5.0984532 | 4.9278965 |
| *GADb* | BGI_novel_G009175 | 3.6633446 | 2.7092906 | 4.3455383 | 4.5465858 |
| *PDP63b* | CSA014790 | 3.2600257 | 2.397803 | 4.7724136 | 4.4073528 |
| *PDH-E1β1* | CSA007666 | 4.0652276 | 3.5096958 | 5.8285808 | 5.3940339 |
| *AQP24a* | CSA023001 | 3.7202785 | 3.2794713 | 5.7527486 | 5.2902032 |
| *PDP60a* | CSA033992 | 3.3391374 | 3.7235586 | 6.2311252 | 5.3262497 |
| *AQP21* | CSA017078 | 4.1358632 | 3.6064422 | 6.4267679 | 5.2265085 |
| *AS3* | CSA000266 | 4.2562559 | 3.9354597 | 5.0849147 | 4.3785116 |
| *PKcyt1* | CSA019642 | 4.3666719 | 3.8698714 | 5.3341392 | 4.9169543 |
| *PDP60b* | CSA001205 | 3.4046307 | 4.0678108 | 5.351911 | 4.5197935 |
| *GDH2b* | CSA023959 | 3.6690268 | 3.7897293 | 5.7183616 | 5.0179219 |
| *PDH-E1β2* | CSA032487 | 3.9107327 | 3.7015491 | 5.6226376 | 4.6932081 |
| *PDP60c* | CSA032532 | 5.0457051 | 4.6870607 | 5.1815002 | 5.0535458 |
| *AQP25* | CSA028896 | 4.6769444 | 4.5801455 | 5.4029268 | 4.117695 |
| *PDH-E2-4a* | CSA004322 | 5.2509616 | 5.0413306 | 3.9882302 | 4.0883112 |
| *PKchlG1* | BGI_novel_G002558 | 4.9283703 | 4.4873577 | 4.3132459 | 4.866908 |
| *AMTb* | CSA022611 | 4.6870607 | 4.4867144 | 4.186659 | 4.4867144 |
| *AQP17* | CSA018859 | 5.7183616 | 5.4809113 | 6.5455051 | 4.6164753 |
| *PEPC1a* | CSA032885 | 6.3264295 | 6.1622906 | 5.4792952 | 5.7161678 |
| *NADH-GOGATb* | CSA027515 | 6.0489772 | 5.2982917 | 5.3183168 | 5.5147535 |
| *PDH-E1α3* | CSA000632 | 5.6313954 | 5.314334 | 4.9453268 | 5.0356239 |
| *NR* | CSA020650 | 4.4269356 | 5.0776703 | 2.4828483 | 4.8752886 |
| *AQP12b* | CSA011041 | 5.0908534 | 5.5153845 | 2.5434959 | 3.960697 |
| *GSc* | CSA001114 | 7.0434101 | 7.4244183 | 4.0609121 | 4.2555007 |
| *PDH-E1α4* | CSA004843 | 6.8284539 | 6.4342946 | 4.8504994 | 4.9040023 |
| *AQP12a* | CSA030470 | 5.9200551 | 6.3428747 | 4.1691233 | 4.7484612 |
| *AQP11a* | CSA036134 | 4.0513721 | 5.0539802 | 9.3325513 | 8.2572464 |
| *AQP11b* | CSA015173 | 2.9183862 | 4.1953476 | 9.2017074 | 7.8378802 |
| *PDP63a* | CSA024803 | 6.3687683 | 4.5484366 | 7.5800701 | 7.1122831 |
| *AQP20a* | CSA033698 | 5.7292808 | 6.1534 | 6.5765221 | 5.9664766 |
| *PDC3a* | CSA002466 | 5.9590746 | 5.4734622 | 6.6623479 | 6.7012718 |
| *PDH-E1α1* | BGI_novel_G002880 | 6.3570241 | 6.1294891 | 7.1344263 | 6.7343032 |
| *NADH-GOGATa* | CSA029477 | 4.2372578 | 4.1268077 | 6.6570683 | 7.3735612 |
| *ALT2a* | CSA034512 | 4.550285 | 3.9221978 | 6.3389592 | 6.7732052 |
| *GADa* | CSA025313 | 5.1168638 | 4.4956952 | 5.7175393 | 6.2110122 |
| *AQP20b* | CSA033696 | 5.2997575 | 5.1771205 | 6.1723275 | 5.5188498 |
| *PDH-E1α2* | BGI_novel_G002249 | 5.1101962 | 4.7876414 | 6.5753123 | 5.9516344 |
| *AQP15b* | CSA024539 | 4.7009945 | 5.2960903 | 6.5938019 | 6.2785424 |
| *NiR* | CSA003866 | 3.8816646 | 5.3359262 | 5.8740592 | 6.4907306 |
| *mtPDKa* | CSA006579 | 4.3785116 | 4.2426025 | 6.649328 | 5.57107 |
| *AQP15c* | BGI_novel_G004130 | 4.2349611 | 4.4215599 | 6.2946207 | 5.4322913 |
| *AMT1-1* | CSA023343 | 4.0214797 | 4.550285 | 5.6636293 | 5.1703257 |
| *SAMDCc* | CSA029628 | 4.7912931 | 4.6982185 | 5.603478 | 5.5734957 |
| *PDH-E2-2* | CSA033762 | 4.7446993 | 4.3589588 | 5.943687 | 5.3899109 |
| *AQP6a* | CSA000157 | 2.4114262 | 1.2927817 | 9.0054842 | 8.9960497 |
| *GDH2a* | CSA011735 | 0.7655347 | 0.7990873 | 10.008429 | 8.7919768 |
| *AS1a* | CSA035384 | 0.8559897 | -0.304006 | 9.3630615 | 7.7613518 |
| *AMTa* | CSA028289 | -0.943416 | -1.785875 | 4.3659724 | 5.3455383 |
| *GLR2.1* | CSA006188 | -2.251539 | -3.321928 | 4.5545889 | 1.722466 |
| *AQP6b* | CSA014714 | -2.736966 | -3.184425 | 6.5169607 | 3.4276062 |
| *PEPC2d* | CSA019990 | 3.4019035 | 3.3377111 | -1.217591 | -1.286304 |
| *PEPC4a* | CSA005647 | 3.397803 | 2.2660369 | -0.473931 | -1.395929 |
| *GLR3.4b* | CSA026127 | 4.33985 | 4.1723275 | 0.0285692 | -0.915936 |
| *PKcyt3* | CSA011300 | 3.1826923 | 3.2883586 | -1 | -0.058894 |
| *AMT1-3* | CSA018541 | 3.6158871 | 3.6299394 | 0.0703893 | -0.029146 |
| *pAOb* | CSA009339 | 5.5567359 | 5.827565 | 3.502076 | 0.0840643 |
| *AQP23* | CSA030184 | 6.5596448 | 6.4517057 | 1.9927684 | -3.836501 |
| *ALT2b* | CSA034511 | -3.643856 | -3.184425 | -0.862496 | -3.058894 |
| *PDH-E1β5* | CSA017019 | -3.643856 | -3.643856 | -2.120294 | -4.321928 |
| *AMT2* | CSA011804 | -4.643856 | -3.473931 | 1.448901 | 0.6780719 |
| *PDH-E1β4* | BGI_novel_G008393 | -5.643856 | -4.643856 | -0.888969 | -1.251539 |
| *PDP42a* | BGI_novel_G004020 | 1.0635029 | 0.2141248 | 3.4235782 | 2.8379432 |
| *PEPC3* | CSA011564 | 1.9145645 | 0.8559897 | 3.2764967 | 3.714795 |
| *PKcyt2* | CSA012967 | 1.2630344 | 1.0285692 | 2.9411063 | 3.1715271 |
| *NRT2.7* | BGI_novel_G005965 | 1.4802651 | 0.1634987 | 1.5310695 | 2.2418402 |
| *pAOc* | CSA015998 | 1.9183862 | 1.1243281 | 2.2234226 | 2.1309309 |
| *PEPC2a* | CSA009904 | 1.8914192 | 1.5058909 | 2.7355222 | 2.3305584 |
| *AMT3-3* | CSA031722 | 0.6599246 | 0.1634987 | 2.9467309 | 0.5459684 |
| *PEPC2c* | CSA035280 | 0.5360529 | 0.1763228 | 1.6690268 | 1.5704629 |
| *PKchl4* | CSA034624 | 0.6507646 | -0.234465 | 1.1505597 | 1.0565835 |
| *PDP38* | CSA014860 | 1.169925 | 0.2387869 | 1.077243 | 1.2809563 |
| *PEPC1b* | CSA019988 | 0.790772 | 0.5655972 | 1.7398481 | 0.2387869 |
| *ASS* | BGI_novel_G008856 | 0.6322682 | 0.7484612 | 0.9560567 | 0.4541759 |
| *PDC3b* | CSA026454 | 0.5459684 | 0.2868811 | 0.7136958 | 0.8073549 |
| *GLR3.7b* | CSA026128 | 1.4329594 | 1.8953026 | -0.074001 | 0.2630344 |
| *GLR3.3b* | CSA020347 | 2.592158 | 2.2479275 | 0.2387869 | 1.3617684 |
| *AQP10* | CSA027609 | 2.6158871 | 1.7311832 | 0.3785116 | 1.4802651 |
| *pAOd* | CSA034879 | 2.1440464 | 1.469886 | 0.8639385 | 1.0565835 |
| *AQP11d* | BGI_novel_G007769 | 1.9963887 | 0.641546 | 1.0976108 | 1.304511 |
| *AS1b* | CSA015297 | 1.9963887 | 1.7004397 | 1.8599695 | 1.7004397 |
| *GLR3.7a* | CSA005105 | 2.5849625 | 2.4329594 | 1.5360529 | 1.3103401 |
| *GMPSb* | CSA002218 | 0.3103401 | 1.0635029 | -1.120294 | -1.943416 |
| *PEPC4b* | CSA005645 | 2.0976108 | 1.4329594 | -0.971431 | -1.888969 |
| *pAOe* | CSA015927 | 1.2326608 | 0.9560567 | -2.120294 | -0.666576 |
| *pAOf* | CSA016220 | 1.1826923 | 0.8559897 | -2.836501 | -0.043943 |
| *AS1c* | CSA010249 | -0.599462 | -0.058894 | -1.358454 | -0.971431 |
| *AQP9b* | BGI_novel_G000041 | 0.0143553 | 0.4329594 | -2.736966 | -1.321928 |
| *AMT3-1c* | CSA023775 | -2 | -2 | -0.358454 | 0.7990873 |
| *PEPC2b* | CSA032884 | -1.599462 | -0.666576 | 2.4594316 | -0.943416 |
| *PDP23b* | CSA000576 | -0.68966 | -0.0145 | -0.395929 | 0.3673711 |
| *ALT1* | BGI_novel_G002945 | 0.1763228 | 0.2141248 | 0.1634987 | -0.666576 |
| *GLR3.4a* | BGI_novel_G002788 | 0.1375035 | -0.736966 | 0.3334237 | 0.1890338 |
| *PKcyt4* | BGI_novel_G006775 | 1.6825733 | 2.0036022 | -2.058894 | -23.2535 |
| *PKchl2* | CSA025782 | 2.4620523 | 1.803227 | -23.2535 | -0.473931 |
| *CuAO* | CSA010475 | -23.2535 | -23.2535 | -1.321928 | -23.2535 |
| *AQP24b* | CSA016542 | -23.2535 | -23.2535 | 2.2690331 | -23.2535 |
| *NRT2.5c* | CSA012405 | -23.2535 | -2.395929 | -2.943416 | -1.152003 |
| *AMT3-1d* | CSA036049 | -23.2535 | -23.2535 | -2.643856 | -1.556393 |
| *GLR3* | CSA002720 | -23.2535 | -23.2535 | 1.0496308 | -2.643856 |
| *AQP20c* | CSA033695 | -23.2535 | -23.2535 | -0.643856 | 0.5160151 |
| *GADd* | CSA004789 | -23.2535 | -23.2535 | 0.5360529 | -0.089267 |
| *GLR2.7* | BGI_novel_G009926 | -23.2535 | -23.2535 | 0.641546 | -0.340075 |
| *AMT3-1b* | CSA022902 | -23.2535 | -23.2535 | 0.9259994 | 0.5360529 |
| *PDH-E2-4b* | BGI_novel_G006357 | -23.2535 | -23.2535 | 1.6644828 | 0.2986583 |
| *AQP19b* | CSA004835 | -23.2535 | -23.2535 | 1.6735564 | 0.5160151 |
| *SAMDCb* | CSA012900 | -23.2535 | -23.2535 | 6.9615076 | 3.6005076 |
| *AMT3-1a* | CSA025550 | -23.2535 | -23.2535 | 2.5680321 | 1.077243 |
| *GLR2.8* | BGI_novel_G006089 | -23.2535 | -23.2535 | 2.8698714 | 3.1440464 |
| *NRT2.5b* | CSA011051 | -23.2535 | -23.2535 | 2.2016339 | 2.989139 |
| *GLR2.2* | CSA026178 | -23.2535 | -23.2535 | 2.2265085 | 2.4956952 |
| *PDH-E2-5b* | CSA011341 | -3.836501 | -3.836501 | -23.2535 | -23.2535 |
| *AMT3.1c* | BGI_novel_G009982 | -5.058894 | -23.2535 | -23.2535 | -23.2535 |
| *Fd-GOGAT* | CSA027086 | -2.473931 | -23.2535 | -23.2535 | -23.2535 |
| *PDC2c* | CSA026455 | -0.68966 | -23.2535 | -23.2535 | -23.2535 |
| *PDC1c* | CSA001947 | -23.2535 | -23.2535 | -23.2535 | -3.643856 |
| *PDC2b* | CSA034822 | -23.2535 | -23.2535 | -23.2535 | -2.643856 |
| *PEPC2e* | CSA019989 | -2.836501 | -23.2535 | -23.2535 | -0.68966 |
| *AQP20d* | CSA033697 | -1.286304 | -23.2535 | -23.2535 | -2.058894 |

**Table S11** The correlation between theanine contents and expression levels of genes involved in theanine metabolism.

| Gene name | Correlation coefficient | Gene name | Correlation coefficient | Gene name | Correlation coefficient | Gene name | Correlation coefficient | Gene name | Correlation coefficient | Gene name | Correlation coefficient |
| --- | --- | --- | --- | --- | --- | --- | --- | --- | --- | --- | --- |
| *NR* | -0.076 | *GADc* | 0.952* | *PEPC1a* | -0.874 | *AMT3.1b* | -0.952* | *PDP23c* | 0.277 | *AQP9a* | 0.597 |
| *NiR* | 0.992* | *GADd* | 0.782 | *PEPC1b* | 0.053 | *AMT3.1c* | -0.770 | *PDP25* | 0.199 | *AQP9b* | -0.730 |
| *GSa* | -0.329 | *AS1a* | 0.565 | *PEPC3* | 0.895 | *AMTa* | 0.954* | *PDP42a* | 0.732 | *AQP10* | -0.777 |
| *GSb* | 0.402 | *AS1b* | -0.651 | *PEPC2a* | 0.625 | *AMTb* | -0.608 | *PDP42b* | -0.430 | *AQP11a* | 0.668 |
| *GSc* | -0.821 | *AS1c* | -0.556 | *PEPC2b* | 0.303 | *AMT3-3* | 0.279 | *PDP42c* | -0.778 | *AQP11b* | 0.607 |
| *GSd* | -0.583 | *AS3* | 0.403 | *PEPC2c* | 0.834 | *AMT3-1a* | 0.588 | *PDP38* | 0.353 | *AQP11c* | -0.774 |
| *NADH-GOGATa* | 0.959* | *GMPSa* | 0.748 | *PEPC2d* | -0.916 | *AMT3-1b* | 0.926 | *PDKa* | 0.572 | *AQP11d* | -0.488 |
| *NADH-GOGATb* | -0.621 | *GMPSb* | -0.722 | *PEPC2e* | 0.622 | *AMT3-1c* | 0.917 | *PDKb* | 0.395 | *AQP12a* | -0.738 |
| *NADH-GOGATc* | -0.719 | *GLR2.1* | 0.398 | *PEPC4a* | -0.956* | *AMT3-1d* | 0.952* | *PDH-E1α1* | 0.619 | *AQP12b* | -0.705 |
| *Fd-GOGAT* | -0.770 | *GLR3.3a* | 0.801 | *PEPC4b* | -0.969* | *NtrB* | -0.788 | *PDH-E1α2* | 0.649 | *AQP13a* | 0.660 |
| *GDH2a* | 0.644 | *GLR3.3b* | -0.843 | *PKa* | 0.863 | *PDC3a* | 0.814 | *PDH-E1α3* | -0.909 | *AQP13b* | 0.575 |
| *GDH2b* | 0.691 | *GLR2.8* | 0.937 | *PKb* | 0.759 | *PDC3b* | 0.731 | *PDH-E1α4* | -0.951* | *AQP15a* | 0.750 |
| *GDH1* | 0.828 | *GLR3.6* | -0.943 | *PKcyt1* | 0.645 | *PDC1a* | 0.919 | *PDH-E1β1* | 0.751 | *AQP15b* | 0.848 |
| *ALT2a* | 0.927 | *GLR2.2* | 0.937 | *PKcyt2* | 0.927 | *PDC1b* | -0.481 | *PDH-E1β2* | 0.556 | *AQP15c* | 0.629 |
| *ALT2b* | 0.346 | *GLR3.7a* | -0.964* | *PKcyt3* | -0.876 | *PDC1c* | 0.764 | *PDH-E1β3* | -0.631 | *AQP17* | -0.135 |
| *ALT1* | -0.774 | *GLR3.7b* | -0.734 | *PKcyt4* | -0.859 | *PDC2a* | -0.689 | *PDH-E1β4* | 0.845 | *AQP19a* | 0.961* |
| *ADCa* | 0.707 | *GLR3* | 0.349 | *PKchlG1* | -0.169 | *PDC2b* | 0.764 | *PDH-E1β5* | 0.119 | *AQP19b* | 0.658 |
| *ADCb* | 0.855 | *GLR2.7* | 0.698 | *PKchlG2* | -0.441 | *PDC2c* | -0.770 | *PDH-E2-1* | 0.740 | *AQP20a* | 0.391 |
| *SAMDCa* | 0.823 | *GLR3.4a* | 0.367 | *PKchlA* | -0.079 | *PDP63a* | 0.620 | *PDH-E2-2* | 0.632 | *AQP20b* | 0.461 |
| *SAMDCb* | 0.367 | *GLR3.4b* | -0.934 | *PKchl4* | 0.623 | *PDP63b* | 0.740 | *PDH-E2-4a* | -0.934 | *AQP20c* | 0.938 |
| *SAMDCc* | 0.877 | *pAOa* | -0.678 | *PKchl2* | -0.915 | *PDP63c* | 0.746 | *PDH-E2-4b* | 0.613 | *AQP20d* | -0.331 |
| *NRT2.5a* | 0.597 | *pAOb* | -0.887 | *ASSchl* | -0.263 | *PDP60a* | 0.670 | *PDH-E2-5a* | -0.913 | *AQP21* | 0.535 |
| *NRT2.5b* | 0.952* | *pAOc* | 0.518 | *ASS* | -0.132 | *PDP60b* | 0.623 | *PDH-E2-5b* | -0.906 | *AQP23* | -0.930 |
| *NRT2.5c* | 0.868 | *pAOd* | -0.884 | *AMT1-1* | 0.755 | *PDP60c* | 0.343 | *AQP3* | -0.934 | *AQP24a* | 0.762 |
| *NRT2.7* | 0.669 | *pAOe* | -0.884 | *AMT1-3* | -0.905 | *PDP60d* | 0.223 | *AQP6a* | 0.904 | *AQP24b* | 0.282 |
| *GADa* | 0.858 | *pAOf* | -0.780 | *AMT2* | 0.747 | *PDP23a* | 0.755 | *AQP6b* | 0.383 | *AQP25* | -0.052 |
| *GADb* | 0.791 | *CuAO* | 0.282 | *AMT3.1a* | -0.844 | *PDP23b* | 0.767 | *AQP7* | 0.755 | *AQP26* | 0.796 |

^*^ indicates significance at *P* < 0.05.
